# Exogenous abscisic acid induces the lipid and flavonoid metabolism of tea plants under drought stress

**DOI:** 10.1038/s41598-020-69080-1

**Published:** 2020-07-23

**Authors:** Zhongshuai Gai, Yu Wang, Yiqian Ding, Wenjun Qian, Chen Qiu, Hui Xie, Litao Sun, Zhongwu Jiang, Qingping Ma, Linjun Wang, Zhaotang Ding

**Affiliations:** 1 Tea Research Institute, Qingdao Agricultural University, Qingdao, 266109 China; 2 College of Life Science, Yantai University, Yantai, 264005 Shandong China; 3 College of Agriculture, Liaocheng University, Liaocheng, 252059 Shandong China; 4 Fruit Tea Station of Weihai Agricultural and Rural Affairs Service Center, Weihai, 264200 Shandong China

**Keywords:** Plant molecular biology, Plant stress responses

## Abstract

Abscisic acid (ABA) is an important phytohormone responsible for activating drought resistance, but the regulation mechanism of exogenous ABA on tea plants under drought stress was rarely reported. Here, we analyzed the effects of exogenous ABA on genes and metabolites of tea leaves under drought stress using transcriptomic and metabolomic analysis. The results showed that the exogenous ABA significantly induced the metabolic pathways of tea leaves under drought stress, including energy metabolism, amino acid metabolism, lipid metabolism and flavonoids biosynthesis. In which, the exogenous ABA could clearly affect the expression of genes involved in lipid metabolism and flavonoid biosynthesis. Meanwhile, it also increased the contents of flavone, anthocyanins, flavonol, isoflavone of tea leaves under drought stress, including, kaempferitrin, sakuranetin, kaempferol, and decreased the contents of glycerophospholipids, glycerolipids and fatty acids of tea leaves under drought stress. The results suggested that the exogenous ABA could alleviate the damages of tea leaves under drought stress through inducing the expression of the genes and altering the contents of metabolites in response to drought stress. This study will be helpful to understand the mechanism of resilience to abiotic stress in tea plant and provide novel insights into enhancing drought tolerance in the future.

## Introduction

Drought is a major abiotic stress for tea plant, it not only limits the productivity of tea plants but also affects the quality of teas^1^. It is essential for us to explore appropriate ways to mitigate the influences of drought stress on tea plants. At present, the research on the impacts of drought stress in tea plants mainly focused on the mechanisms underlying the stress response, which included morphological, physiological and molecular changes^1–3^.

The physiological changes, including the contents of chlorophyll, proline, MDA and the activities of antioxidant enzymes, have been extensively investigated in tea plants under drought stress^4,5^. A large number of drought-inducible genes and proteins in tea plants have also been identified by transcriptomic and proteomic analysis^3,6,7^. A former study in our lab showed that a large numbers of differentially expressed genes (DEGs) in response to drought stress were mainly enriched in volatile compounds, flavonoids, theanine biosynthesis pathways, and some DEGs were also involved in leaf senescence, such as lipoxygenase (*LOX*), salicylate/benzoate carboxyl methyltransferase (*BSMT*), arginine decarboxylase (*ADC*), glutamine synthetase (*GS*), glutaminase (*GLS*), chalcone isomerase (*CHI*), flavonoid 3′,5′-hydroxylase (*F3′5′H*), flavonol synthase (*FLS*), flavanone 3-hydroxylase (*F3H*)^1^. Another study in our lab showed that a large numbers of the differentially expressed proteins in response to drought stress were mainly involved in glycolysis/gluconeogenesis, starch and sucrose synthesis, or degradation metabolism, and second metabolism^6^. Recent research in our lab found that many lysine ubiquitination proteins (Kub proteins) related to catechins biosynthesis (e.g., PAL, CHS, CHI and F3H), carbohydrate and amino acid metabolism (e.g., FBPase, FBA and GAD1), were significantly induced by drought stress, suggesting that these Kub proteins might affect the degradation of proteins, the synthesis of catechins, and the accumulation of sucrose, fructose and GABA in tea leaves^7^.

Abscisic acid (ABA) is an important phytohormone responsible for activating drought resistance. Drought stress induces ABA biosynthesis, which is the signal that triggers a number of molecular and cellular responses, ultimately resulting in stomatal closure. Several studies indicated that ABA could enhance stress tolerance either as a result of its endogenous concentration or through exogenous application^8,9^. For instance, the exogenous ABA is beneficial for plants to enhance drought resistance, as it induces stomatal closure, osmotic adjustment, and increases activities of antioxidant enzymes in tomato, spring wheat and *Populus*^5,10,11^. Moreover, the exogenous ABA could improve protein transport, carbon metabolism and expression of resistance proteins to enhance drought tolerance of tea plants^12^. However, to our knowledge, the regulation mechanism of exogenous ABA on genes and metabolites of tea plants under drought stress was rarely reported. In the present study, we analyzed the effects of exogenous ABA on genes and metabolites of tea leaves under drought stress using transcriptome and metabolomics. The study will contribute to further an improved understanding of the molecular mechanism of tea plants in response to drought stress.

## Results

### Physiological changes of tea leaves under AT and SD

**Figure 1:**
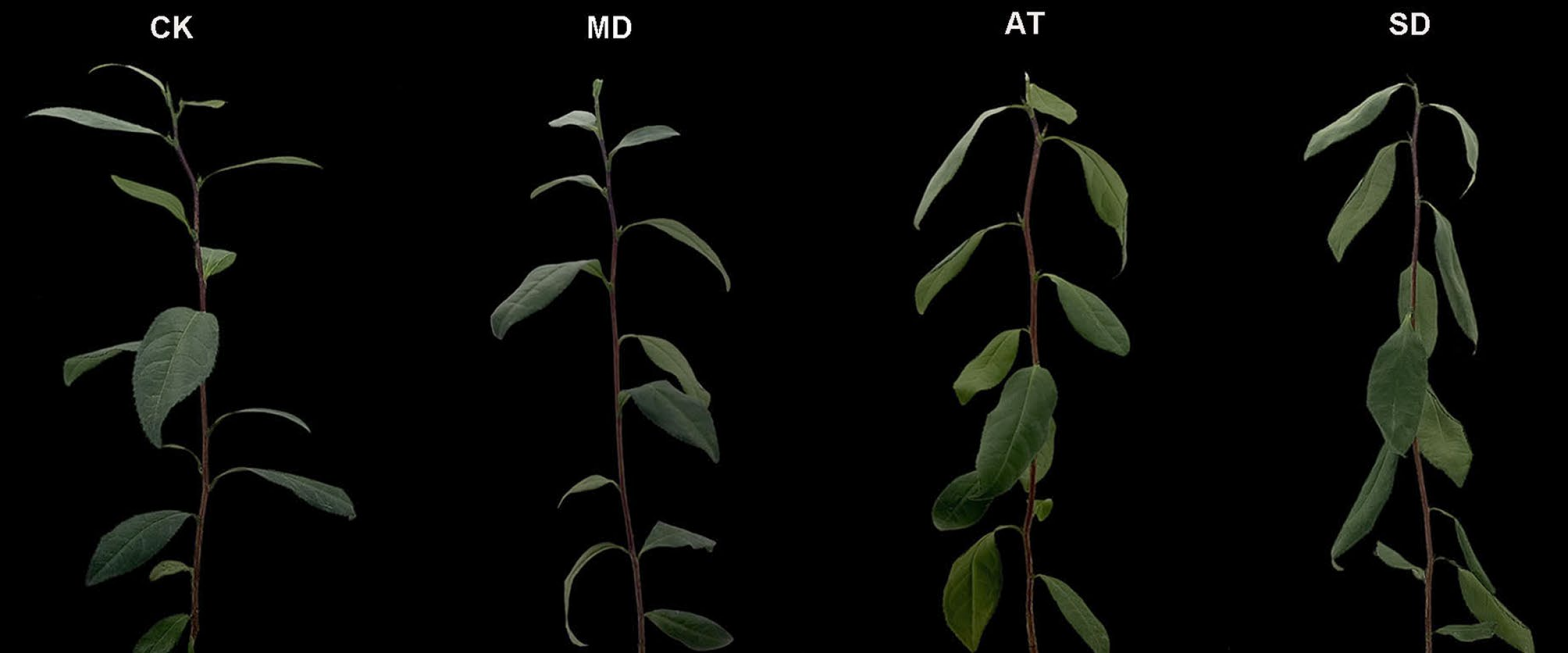
The phenotype changes of tea leaves under CK, MD, AT and SD. The phenotype of CK was photographed under normal growth conditions, the phenotype of MD was photographed with drought for 24 h, the phenotypes of AT and SD were photographed at 53 h after spraying with equal ABA solution and distilled water, respectively.

To investigate the phenotype changes of tea plants treated with exogenous ABA and drought, their stress phenotypes (displayed visible morphological changes) were photographed. The results showed that a number of wilted and curled leaves were observed under SD (Fig. 1). While the wilted and curled leaves were remarkably relieved under AT, indicating that the exogenous ABA partly relieved the damage and maintained the growth of tea plants under drought stress.

To assess the effects of exogenous ABA on physiological characterization of tea leaves, we detected several physiological indexes of tea leaves under well-watered plants (CK), mild drought (MD), ABA treatment (AT) and severe drought (SD). As shown in Table 1, the content of total chlorophyll (TC) and leaf water content (LWC) decreased as the duration of the drought treatment increased under AT and SD, but it was higher under AT than that under SD. The value of maximum quantum yield of PSII (*Fv*/*Fm*) also showed similar changes. The content of MDA increased as the duration of the drought treatment increased under AT and SD, but it was lower under AT than that under SD. The results indicated that the exogenous ABA could reduce lipid peroxidation, prevent the degradation of chlorophyll and maintain photosynthesis of tea leaves under drought stress. In addition, the contents of three endogenous hormones and the activities of four antioxidant enzymes were also measured, the activity of ascorbate peroxidase was significantly lower under AT than that under SD, there was no difference in the activity of CAT, POD and GR. The content of endogenous ABA in tea leaves is significantly higher under AT than that under SD, but the content of endogenous GA_3_ is significantly lower, there was no difference in the content of endogenous IAA. The results suggested that the exogenous ABA could affect the changes of endogenous hormones and antioxidant enzymes of tea leaves under drought stress.

**Table 1:** Physiological indexes of tea leaves during drought stress.

Parameters	CK	MD	AT	SD
MDA (nmol/g)	43.87 ± 0.88 b	46.25 ± 0.88 b	47.54 ± 0.92 ab	51.85 ± 2.01 a
LWC (%)	75.88 ± 1.02	73.23 ± 0.37	68.46 ± 0.7	65.36 ± 0.82
CAT (U/g)	98.48 ± 3.29 a	80.21 ± 1.75 c	82.76 ± 1.72 bc	89.01 ± 1.80 b
POD (U/g)	105.09 ± 3.09 a	110.33 ± 2.63 a	89.17 ± 0.92 b	86.18 ± 0.64 b
APX (μmol/min/g)	1.55 ± 0.05 a	0.6186 ± 0.01 c	0.68 ± 0.02 c	0.9517 ± 0.04 b
GR (μmol/min/g)	483.26 ± 2.81 bc	433.22 ± 16.81 c	536.16 ± 16.96 ab	594.06 ± 25.23 a
ABA (μg/g)	0.4669 ± 0.0194 b	0.533 ± 0.024 b	2.6333 ± 0.031 a	0.4473 ± 0.018 b
IAA (μg/g)	0.6124 ± 0.0113b	0.8129 ± 0.0338a	0.6303 ± 0.0158b	0.5862 ± 0.0124b
GA_3_ (μg/g)	0.495 ± 0.0393 d	0.6711 ± 0.0417 c	0.9108 ± 00,397 b	1.1986 ± 0.0996a
*Fv/Fm*	0.83 ± 0.21 a	0.79 ± 0.19 ab	0.73 ± 0.18 b	0.65 ± 0.57 c
Chl content (mg/g)	3.17 ± 0.33 a	3.01 ± 0.27 ab	2.88 ± 0.12 ab	2.46 ± 0.21 b

The date in the table are represented as the mean ± standard deviation of three biological replicates, lowercase letters indicated statistical significance—samples not sharing a letter differed significantly according to Duncan test at *P* < 0.05.

*CK* control group, *MD* mild drought group, *AT* ABA treatment group, *SD* severe drought group, *MDA* malondialdehyde, *CAT* catalase, *POD* Peroxidase, *APX* ascorbate peroxidase, *GR* glutathione reductase. The above data were determined under CK (0 h), MD (24 h), AT (77 h) and SD (77 h).

### The analysis of transcriptome

To explore the transcript events of exogenous ABA on tea plants under drought, the sample leaves of CK, MD, AT and SD were conducted to RNA-seq analysis. A total of 619,817,958 clean reads were obtained from 12 RNA-Seq libraries. The Q30 percentage were over 92.54%, and the average GC content was over 44.52%. Overall, the results indicated that the RNA-Seq datasets were robust quality and could be used for further analysis (Supplementary Table S1).

**Figure 2:**
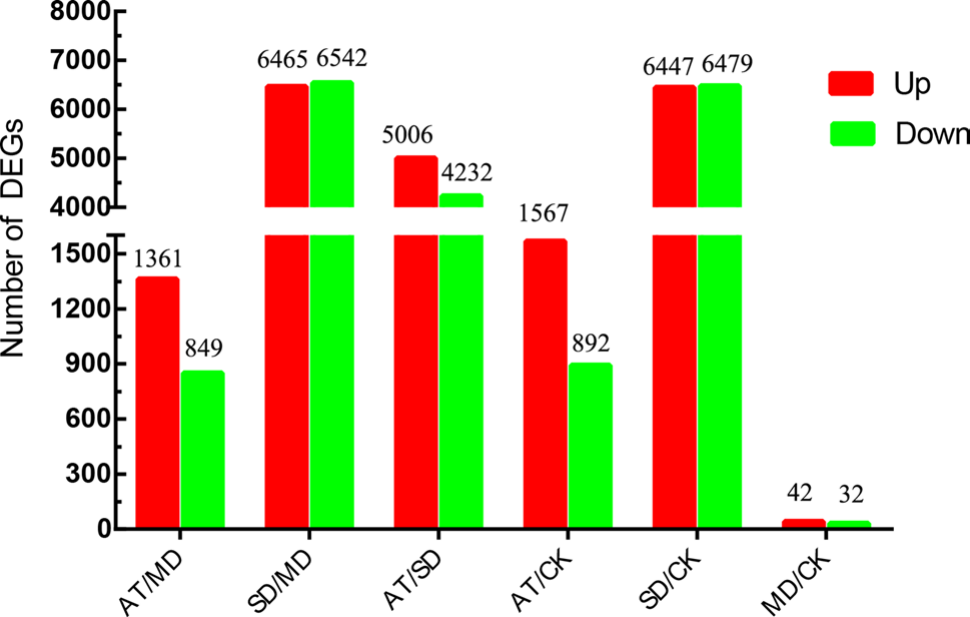
Statistics of the differentially expressed genes (DEGs) between different treatment groups.

A total of 2,210 DEGs (1,361 up- and 849 down-regulated) were obtained in AT/MD, 13,007 DEGs (6,465 up- and 6,542 down-regulated) were obtained in SD/MD. A total of 2,459 DEGs (1,567 up- and 892 down-regulated) were also obtained in AT/CK, 12,926 DEGs (6,447 up- and 6,479 down-regulated) were also obtained in SD/CK. The DEGs were obtained between AT/SD and MD/CK (Fig. 2). The results revealed that exogenous ABA can effectively relieve the drought stress of tea plants and decrease the expression amounts of response genes to drought.

GO analysis of the above DEGs revealed that the highly enriched terms of the biological process category were ‘cellular process’, ‘metabolic process’, ‘biological regulation’ and ‘response to stimulus’. Within the cellular component category, the highly enriched terms were ‘cell’, ‘cell part’ and ‘organelle’. Within the molecular function category, the highly represented terms were ‘binding’, ‘catalytic activity’ and ‘transporter activity’ (Supplementary Fig S1).

KEGG pathway enrichment analysis revealed that the DEGs in AT/MD were mainly enriched in starch and sucrose metabolism, lipid metabolism, plant-pathogen interaction, plant hormone signal transduction. The DEGs in AT/CK were mainly enriched in fructose and mannose metabolism, starch and sucrose metabolism, plant hormone signal transduction, glycerolipid metabolism. The DEGs in SD/MD were mainly enriched in photosynthesis, biosynthesis of amino acids, biosynthesis of secondary metabolites, starch and sucrose metabolism. The DEGs in SD/CK were mainly enriched in photosynthesis, Glycine, serine and threonine metabolism, starch and sucrose metabolism, Phenylalanine, tyrosine and tryptophan biosynthesis. The DEGs in MD/CK were mainly enriched in starch and sucrose metabolism and plant hormone signal transduction. The DEGs in AT/SD were mainly enriched in carbon metabolism, biosynthesis of amino acids, biosynthesis of secondary metabolites (Supplementary Fig S2). KEGG enrichment analyses indicated that the DEGs between CK and other three treatments were mainly enriched in energy metabolism, biosynthesis of amino acids, lipid metabolism and biosynthesis of secondary metabolites.

### The effect of exogenous ABA on genes related to energy metabolism and amino acid metabolism of tea plants under drought stress

**Figure 3:**
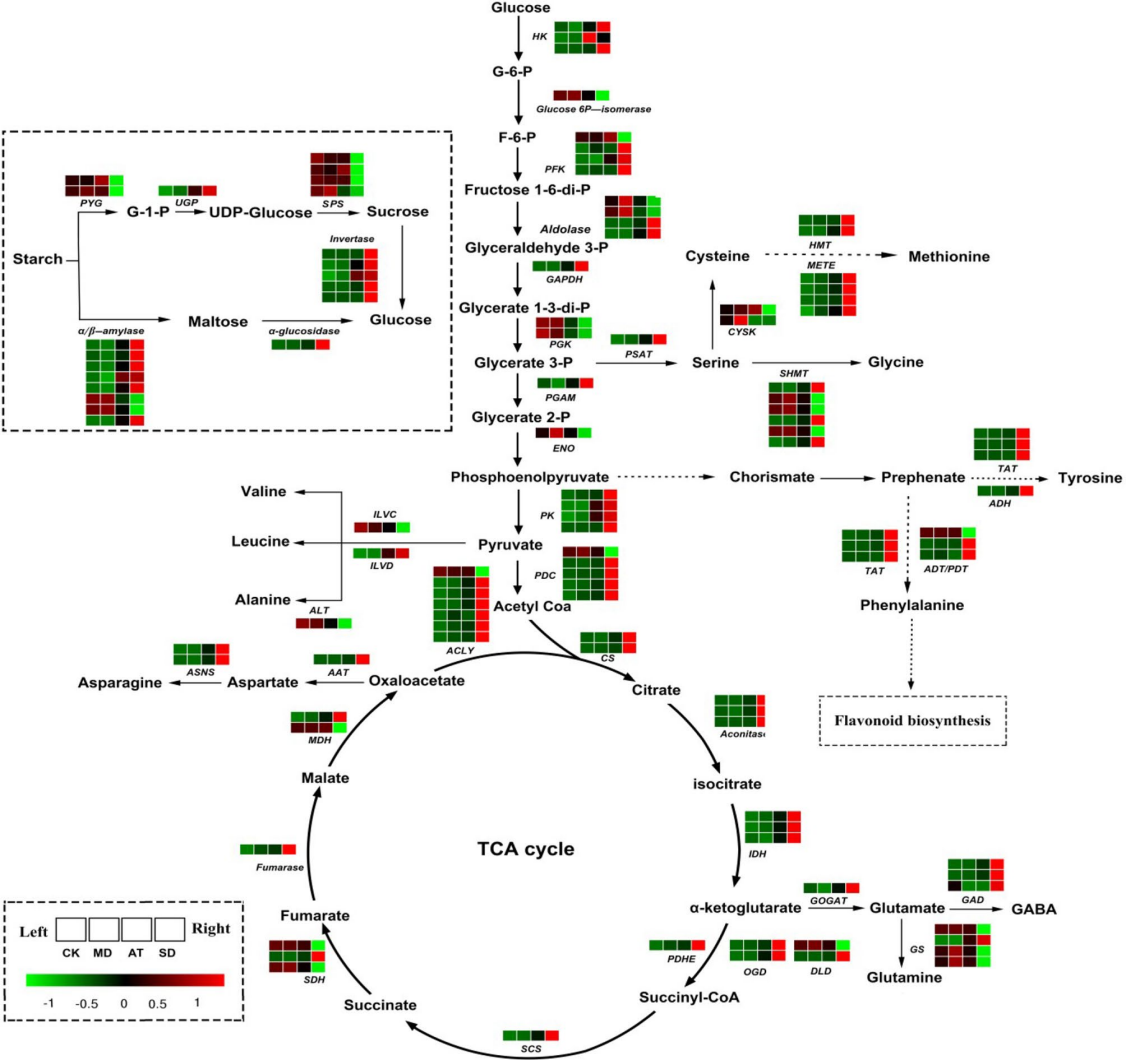
The expression of genes related to starch and sucrose metabolism, glycolysis, TCA cycle and amino acids pathway under CK, MD, AT and SD. HK, hexokinase; G6PI, glucose-6-phosphate; PFK, 6-phosphofructokinase; Aldolase, fructose-bisphosphate aldolase; GAPDH, glyceraldehyde 3-phosphate; PGK, phosphoglycerate kinase; PGAM, phosphoglycerate mutase; ENO, enolase; PK, pyruvate kinase; PDC, pyruvate dehydrogenase; ACLY, ATP citrate synthase; CS, citrate synthase; Aconitase, aconitate hydratase; IDH, isocitrate dehydrogenase; PDHE, pyruvate dehydrogenase; OGD, oxoglutarate dehydrogenase complex; DLD, dihydrolipoamide dehydrogenase; SCS, succinyl-CoA synthetase; SDH, Succinate dehydrogenase; FUM, fumarate hydratase; MDH, malate dehydrogenase; PYG, glycogen phosphorylase; UGP, UTP-glucose-1-phosphate; SUS, sucrose synthase; α-glucosidase, alpha-glucosidase; Invertase, beta-fructofuranosidase; AMY, alpha-amylase; BAM, beta-amylase; GOGAT, glutamate synthase; GAD, glutamate decarboxylase; GS, glutamine synthetase; AAT, aspartate aminotransferase; ASNS, asparagine synthase; ILVC, ketol-acid reductoisomerase; ILVD, dihydroxy-acid dehydratase, ALT, branched-chain amino acid aminotransferase; TAT, tyrosine aminotransferase; ADH, arogenate dehydrogenase; ADT/PDT, arogenate/prephenate dehydratase; PSAT, phosphoserine aminotransferase; SHMT, Serine hydroxymethyltransferase; CYSK, cysteine synthase; HMT, homocysteine S-methyltransferase; METE, 5-methyltetrahydropteroyltriglutamate.

As for starch and sucrose metabolism, the genes involved in starch synthesis were significantly down-regulated in both SD/MD and SD/CK, but unchanged or slightly down-regulated in both AT/MD and AT/CK, including ADP-glucose pyrophosphorylase (*AGPase*), starch synthase (*SS*), granule-bound starch synthase (*GBSS*), starch-branching enzyme (*SBE*). While the starch degradation-related genes were up-regulated in both SD/MD and SD/CK, but unchanged or slightly up-regulated in both AT/MD and AT/CK, including *AMY* (α-amylase) and *BAM* (β-amylase). Similarly, four *SPS* genes (sucrose-phosphate synthase) involved in sucrose synthesis were down-regulated in both SD/MD and SD/CK, but unchanged or slightly down-regulated in both AT/MD and AT/CK. While the sucrose degradation-related genes were obviously up-regulated in both SD/MD and SD/CK, but unchanged or slightly up-regulated in both AT/MD and AT/CK, such as *INV* (invertase) (Fig. 3, Supplementary Table S2).

Regarding the glycolysis and TCA cycle, most of genes involved in glycolysis and TCA cycle were significantly up-regulated in both SD/MD and SD/CK, but unchanged or slightly up-regulated in both AT/MD and AT/CK, including hexokinase (*HK*), pyruvate kinase (*PK*), phosphofructokinase (*PFK*), phosphoglycerate mutase (*PGAM*) in glycolysis; ATP citrate synthase (*ACLY*), citrate synthase (*CS*), aconitate hydratase (*Aconitase*), isocitrate dehydrogenase (*IDH*), succinyl-CoA synthetase (*SCS*) in TCA cycle (Fig. 3, Supplementary Table S2).

Regarding the amino acid metabolism, the expression of most genes was significantly up-regulated in both SD/MD and SD/CK, but unchanged or slightly up-regulated in both AT/MD and AT/CK under drought stress, including tyrosine aminotransferase (*TAT*), arogenate dehydrogenase (*ADH*), glutamate decarboxylase (*GAD*), glutamate synthase (*GOGAT*), asparagine synthase (*ASNS*) (Fig. 3, Supplementary Table S2).

### The effect of exogenous ABA on genes related to lipid metabolism of tea plants under drought stress

To investigate the effect of exogenous ABA on lipid metabolism of tea plants, we mainly analyzed the expression of genes involved in lipid metabolism under drought stress. A total of 81 DEGs related to lipid metabolism were selected, of which 40 DEGs were clearly up-regulated in both SD/MD and SD/CK, but unchanged or slightly up-regulated in both AT/MD and AT/CK, including diacylglycerol kinase (*DGK*), fatty acyl-ACP thioesterase B (*FATB*), ethanolamine kinase (*EKI*), phospholipase D1/2 (*PLD1/2*). In addition, 39 DEGs were significantly down-regulated in both SD/MD and SD/CK, but unchanged or slightly down-regulated in both AT/MD and AT/CK, including lipoxygenase (*LOX2S*), 12-oxophytodienoic acid reductase (*OPR*), phosphatidylserine decarboxylase (*PSD*), phosphatidylserine synthase (*PTDSS*). In addition, the lysophospholipase (*LYPLA*) and linoleate 9S-lipoxygenase (*LOX1_5*) were found to be only up-regulated in both AT/MD and AT/CK.

### The effect of exogenous ABA on genes related to phenylpropanoid and flavonoid metabolism of tea plants under drought stress

**Figure 4:**
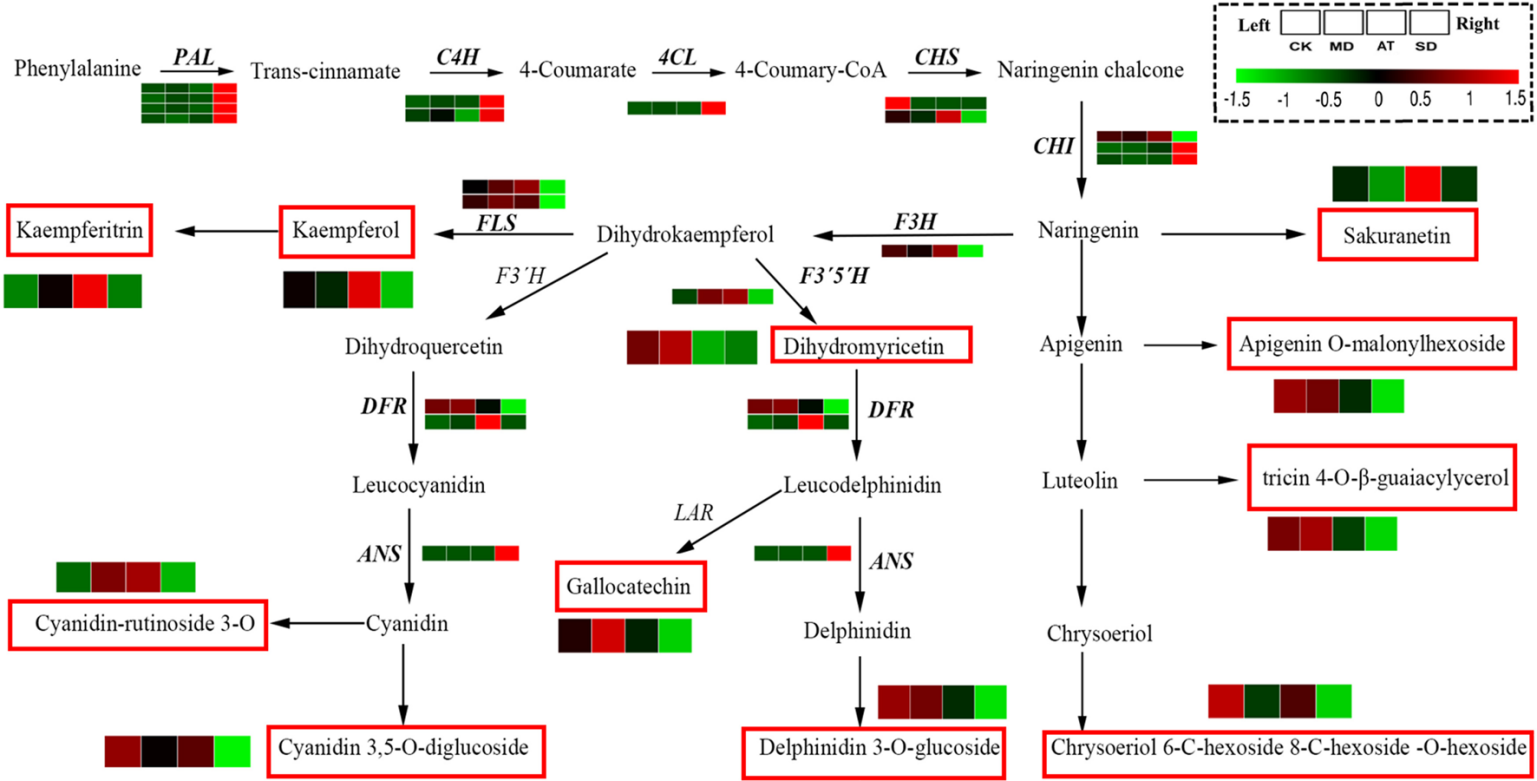
The expression of genes related to phenylpropanoid and flavonoid metabolism under CK, MD, AT and SD. PAL, phenylalanine ammonia-lyase; C4H, cinnamic acid 4-hydroxylase; 4CL, 4-coumarate-CoA ligase; CHS, chalcone synthase; CHI, chalcone isomerase; F3′H, flavonoid 3′-hydroxylase; F3′5′H, Flavonoid 3′,5′-hydroxylase; DFR, Dihydroflavonol 4-reductase; LAR, Leucoanthocyanidin reductase; ANS, leucoanthocyanidin dioxygenase; FLS, flavonol synthase.

To investigate the effect of exogenous ABA on phenylpropanoid and flavonoid metabolism of tea plants, we analyzed the expression of genes involved in phenylpropanoid and flavonoid biosynthesis under drought stress (Fig. 4). The results showed that the vital genes related to phenylpropanoid biosynthesis were highly up-regulated in both SD/MD and SD/CK, but unchanged or slightly up-regulated in both AT/MD and AT/CK, such as cinnamyl-alcohol dehydrogenase (*CAD*), 4-coumarate-CoA ligase (*4CL*), ferulate-5-hydroxylase (*F5′H*), phenylalanine ammonia-lyase (*PAL*). While the key genes related to flavonoid biosynthesis were significantly down-regulated in both SD/MD and SD/CK, but unchanged or slightly down-regulated in both AT/MD and AT/CK, including *F3′H*, *F3′5′H*, *FLS* and *DFR* (dihydroflavonol 4-reductase).

**Figure 5:**
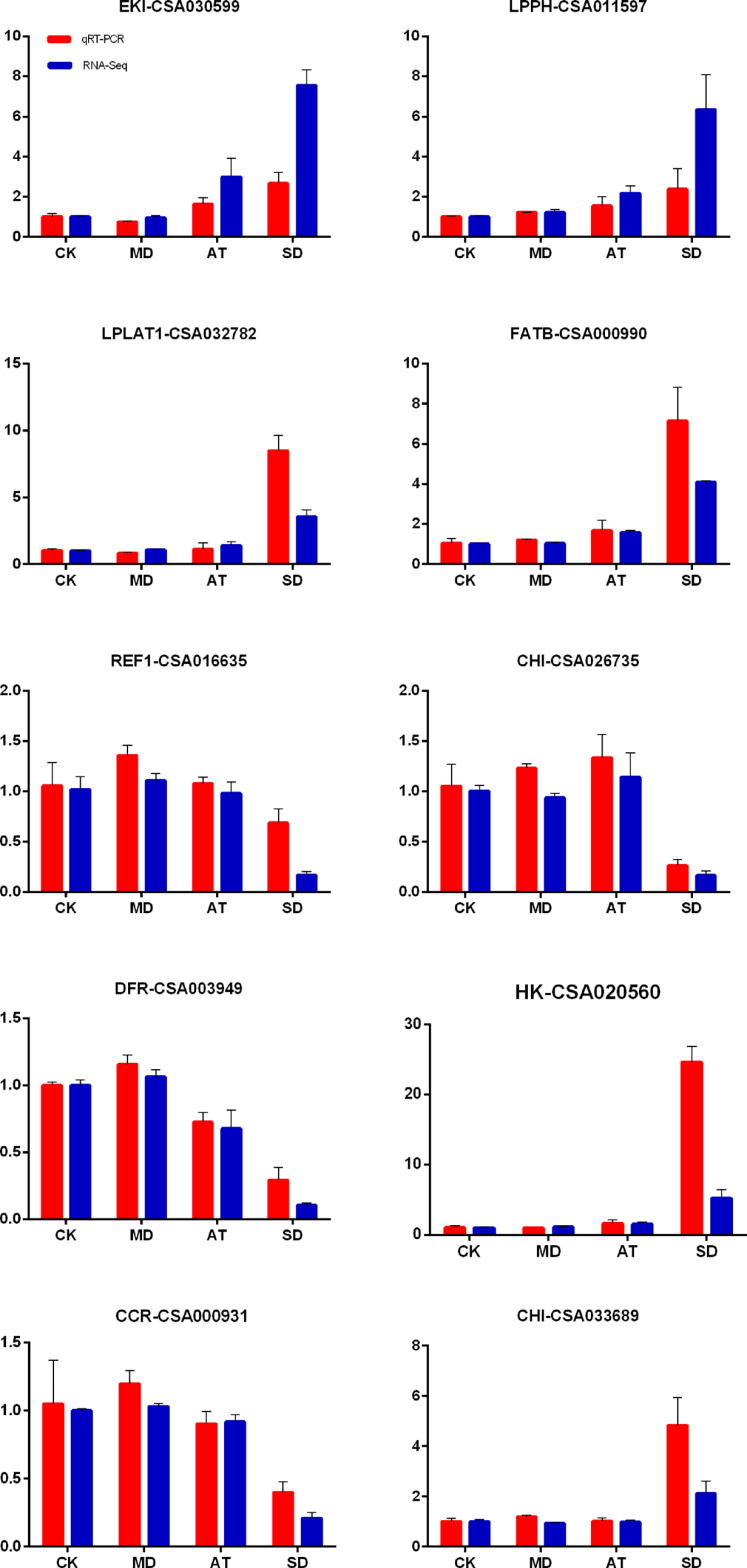
qRT-PCR analysis of a set of DEGs. The expression at CK was set as 1, and the relative expression level was calculated for several genes.

To validate the accuracy and repeatability of transcriptome sequencing data, ten DEGs were randomly selected to validate the RNA-seq data by qRT-PCR (Fig. 5). The results showed the similar expression patterns between RNA-seq and qRT-PCR, suggesting that the RNA-seq data is reliable.

### Metabolic differences of flavonoids and lipid metabolism under AT and SD

**Figure 6:**
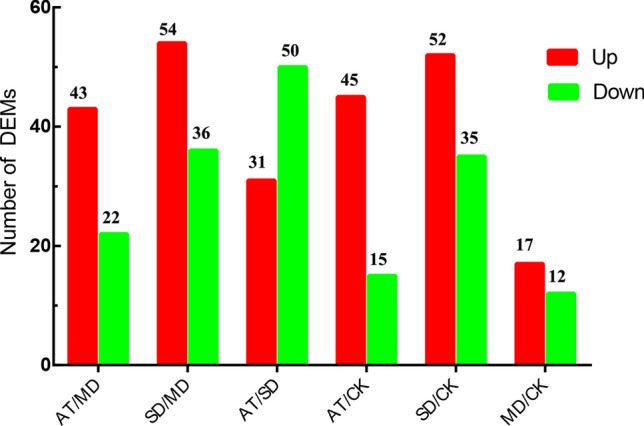
Statistics of the differential metabolites (DEMs) (VIP ≥ 1 and fold change ≥ 2 or ≤ 0.5) between different treatment groups.

To investigate the effects of exogenous ABA on metabolites of tea leaves in response to drought stress, the second fully expanded leaves at the top of CK, MD, AT and SD were conducted to LC–ESI–MS/MS analysis. A total of 65 DEMs (differential metabolites) (43 up- and 22 down-regulated) were obtained in AT/MD, 90 DEMs (54 up- and 36 down-regulated) were obtained in SD/MD, and 81 DEMs (31 up- and 50 down-regulated) were obtained in AT/SD (Fig. 6). Interestingly, the abundances of most flavonoids were markedly increased, while the abundances of most lipid metabolites were markedly decreased in AT/MD compared to those in SD/MD.

For the flavonoids, the most compounds (flavone, flavanone, flavonol, isoflavone and anthocyanins) were obviously increased in AT/MD (Fig. 4). Especially, the abundances of sakuranetin in flavone showed 13.15- and 11.06-fold increase in AT/MD and SD/MD, respectively. The levels of prunetin, acacetin, laricitrin and syringetin were only increased in AT/MD. The levels of apigenin O-malonylhexoside, delphinidin 3-O-glucoside and cyanidin 3-O-rutinoside were significantly decreased in SD/MD. In addition, the levels of kaempferol, kaempferitrin, 4-methylcatechol, cyanin and fustin were higher in AT/MD than that in SD/MD.

For the lipid metabolites (Supplementary Table S3), 38 metabolites involved in fatty acids, glycerolipids and glycerophospholipids metabolisms were markedly changed under drought stress. Especially, LysoPS 22:6 showed higher abundance with 11.57- and 10-fold increase in SD/MD and AT/MD, respectively. The levels of LysoPE 14:0, LysoPE 16:0, LysoPE 18:0, LysoPE 18:1, LysoPE 18:2 (2n isomer), LysoPC 15:1, LysoPC 16:0 and LysoPC 17:0 increased significantly in SD/MD. The levels of PC 16:1/14:1 and 13-HOTrE were only increased in AT/MD. In addition, 13 metabolites were only increase in SD/MD, such as, LysoPC 18:0 (2n isomer), LysoPC 18:2 (2n isomer) and MAG (18:1) isomer2.

### Interaction network analysis between genes and metabolites

Gene-metabolite interaction networks could be used to help understand functional relationship and to aid in identifying new regulatory elements^13^. Here, Pearson correlation tests were carried out between differentially expressed genes and metabolites related to flavonoids and lipid metabolism.

For phenylpropanoid and flavonoid biosynthesis, both 113 DEGs and 22 DEMs related to phenylpropanoid and flavonoids biosynthesis were carried out Pearson correlation analysis. The result showed that 43 DEGs had strong positive and negative correlation coefficient values (R^2^ > 0.8 or < − 0.8 and *P* value < 0.05) with 12 metabolites (Supplementary Table S4). For example, there was significantly positive correlation between the gene expression (*CCR*,* DFR*,* CHI3*,* PER42*) and metabolite abundances (gallocatechin, apigenin O-malonylhexoside, delphinidin 3-O-glucoside), but there was significantly negative correlation between the gene expression (*CAD*) and metabolite abundances (apigenin O-malonylhexoside, gallocatechin, dihydromyricetin and mirtillin).

**Figure 7:**
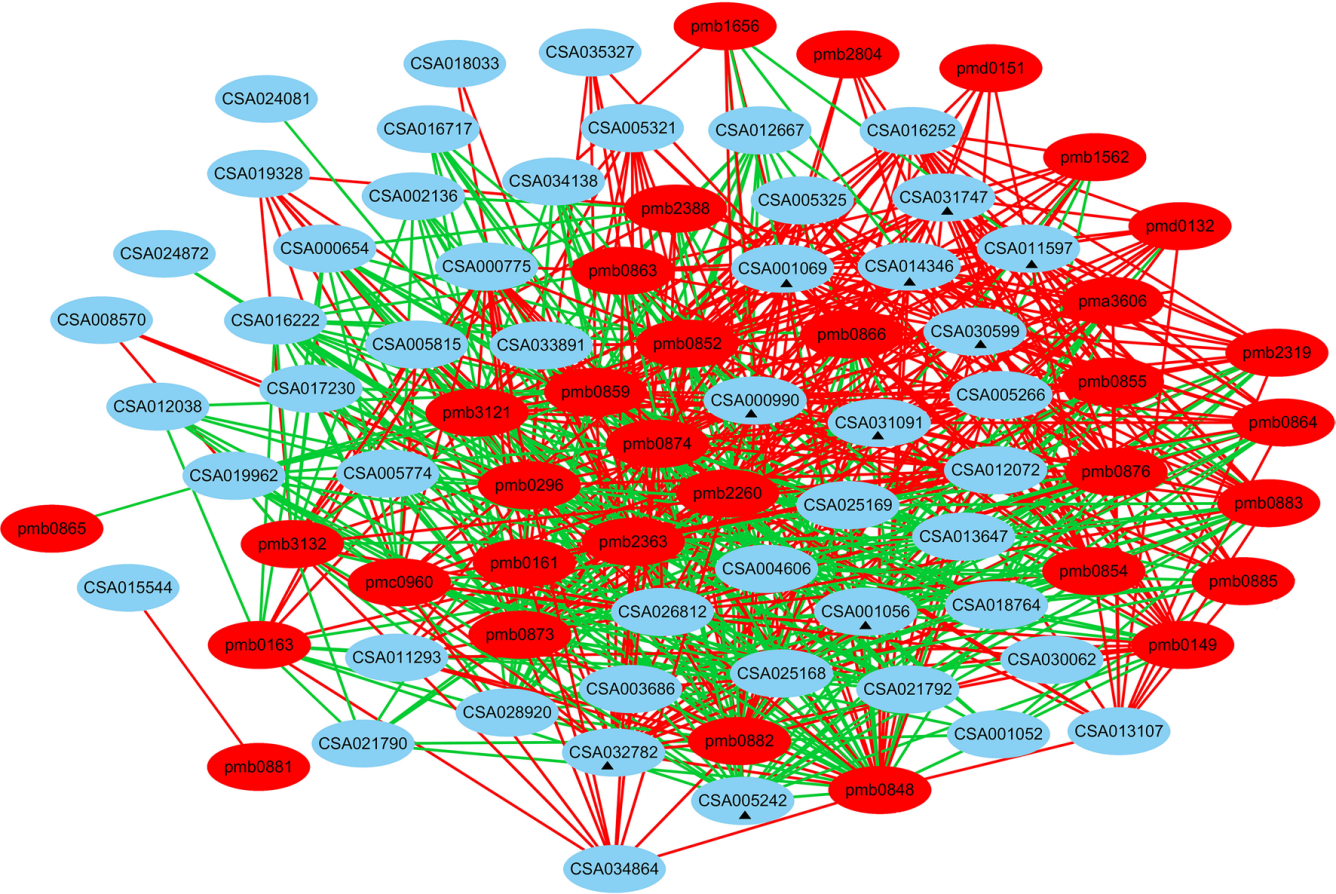
The connection network between genes and metabolites related to lipid metabolism. The red lines indicate positive correlation, the green line indicates negative correlation. Filled triangle indicated that the ten genes were significantly positive or negative correlation between the expression of genes and abundances of most lipid metabolite.

For lipid metabolism, both 81 DEGs and 38 DEMs related to lipid metabolisms were carried out Pearson correlation analysis, the result showed that 56 DEGs had strong positive and negative correlation coefficient values (R^2^ > 0.8 or < − 0.8 and *P* value < 0.05) with 34 metabolites (Fig. 7, Supplementary Table S5). Further analysis indicated that there was significantly positive correlation between the expression of eight key genes and abundances of most lipid metabolite, including diacylglycerol kinase 1 (*DGK1*), soluble epoxide hydrolase (*EPHX2*), fatty acyl-ACP thioesterase B (*FATB*), ethanolamine kinase (*EKI*), diacylglycerol kinase 2 (*DGK2*), acetyl-CoA acyltransferase 1 (*ACAA1*), glutathione peroxidase (*GPX*), lysophospholipid acyltransferase1 (*LPT1*). There was significantly negative correlation between the expression of two genes and abundances of most lipid metabolite, including lysophosphatidylcholine acyltransferase (*LPCAT*) and aldehyde dehydrogenase (*ALDH*).

## Discussion

ABA is a pivotal hormone in plant responses to biotic and abiotic stress, playing a key role in adapting metabolism and gene expression to help plant cope with stress conditions^14^. Previous research in tea plants showed that the exogenous ABA could improve protein transport, carbon metabolism and enhance the expression of resistance proteins (Hsp70) to enhance drought tolerance of tea plants^12^. In the present study, to gain further insight into the effects of exogenous ABA on genes and metabolites of tea plants under drought stress, we integrated transcriptomic and metabonomic analysis to explore the regulation mechanism. The results showed that exogenous ABA could regulate the expression of the genes related to energy metabolism, lipid metabolism and flavonoid biosynthesis to promote energy storages and the balance of primary metabolism, maintain membrane integrity, produce more flavonoids and derivatives in response to drought stress.

### Exogenous ABA promoted energy storages and maintained the balance of primary metabolism

Starch is a key molecule in mediating plant responses to abiotic stresses, such as water deficit. Under drought stress conditions, plants generally remobilize starch to provide energy and carbon at times when photosynthesis is potentially limited^15^. The presence of sufficient sucrose can serve as the important energy source for the cells, and they act as an essential osmoprotectant to protect biomembranes and proteins against abiotic stress^16^. Previous study in tea plants showed that the expression of the genes related to starch synthesis (*AGPase*,* SS*) was inhibited and the expression of the genes related to starch degradation (*AMY*,* BAM*) was induced, whereas the expression of the genes related to sucrose synthesis (*UDPGase*,* SPS*) and degradation (*INVs*) was highly induced under drought stress^1,2^. However, in tea plants, the effect of exogenous ABA on genes involved in starch and sucrose metabolism was rarely reported.

In the present study, transcriptome analysis showed that the genes involved in starch and sucrose synthesis of tea leaves were up-regulated, and those related to starch and sucrose degradation were down-regulated in AT compared with SD (Fig. 3), suggesting that the exogenous ABA might promote a shift in metabolism towards a storage metabolism in tea leaves under drought stress. In addition, the exogenous ABA significantly decreased the expression of genes involved in TCA cycle, glycolysis and amino acid metabolism compared with severe drought. Metabolomic results showed that the exogenous ABA significantly increased the contents of the ketoglutaric acid, succinic acid and citric acid compared with severe drought. The results indicated that the exogenous ABA possible played the important role in promoting the energy storages and maintaining the balance of primary metabolism of tea plants under drought stress.

### Exogenous ABA affected the expression of genes related to lipid metabolism in tea leaves under drought stress

Lipids exert multiple roles and functions in plant tissues: constituents of the cell membrane, storage molecules of metabolic energy, and as signaling factors in response to stressors^17^. Altered lipid biosynthesis, biomembrane rearrangement, and specific fatty acid changes reduce the impairment of cell membranes by abiotic stress^18^. Several studies showed that lipid metabolism in different plants was modified by drought stress^19–22^. In the moss *Atrichum androgynum*, the contents of phosphoglyceride and monogalactosyldiacylglycerol (MGDG) were significantly reduced, but the content of triacylglycerols (TAGs) was significantly increased under drought stress^22^. In peanut, the contents of phospholipids and galactolipids were significantly decreased under drought stress^21^. In addition, several studies showed that ABA could regulate lipid metabolism in different plant species^23,24^. For example, the exogenous ABA on mungbean leaves induced a slight increase in the content of phospholipids and a decrease in the content of free fatty acids, whereas monogalactosyldiacylglycerol (MGDG) content was significantly reduced in N-leaves after application of ABA^24^ . However, in tea plants, the effect of exogenous ABA on genes and metabolites related to lipid metabolism in response to drought stress was rarely reported.

In this work, transcriptome analysis showed that the expression of genes involved in lipid metabolism of tea leaves under drought stress was regulated by exogenous ABA. For example, the expression of the *FATB*,* EKI*, *ACAA1*, *DGK* and *GPX* related to lipid biosynthesis was significantly down-regulated and the expression of the *LPCAT* and *ALDH* related to lipid degradation (CSA005242, CSA012038) was significantly up-regulated by exogenous ABA (Supplementary Table S5). Aldehyde dehydrogenases (*ALDH*s) play a major role in the detoxification processes of aldehydes generated in plants when exposed to abiotic stress^25^. Enhancing antioxidant defense seems to be one of the approaches for plants to reduce the level of oxidative stress. Various plant *ALDH* genes have been reported to be activated by environmental stress including drought and salinity^26,27^, for example, Two *ALDH* genes from *Arabidopsis* and barley were shown to be up-regulated by drought stress for improving drought tolerance^28,29^. Previous studies showed that transgenic tobacco and *Arabidopsis* plants overexpressing *ALDHs* showed elevated tolerance against oxidative stress, which was correlated with decreased accumulation of reactive oxygen species and malondialdehyde derived from cellular lipid peroxidation^30,31^. It was reported previously that ABA treatment strong induced the expression of *ALDH7B4* in *Arabidopsis thaliana*^32^. Therefore, we speculated that up-regulated expression of *ALDHs* induced by ABA might improve stress tolerance most likely by scavenging toxic aldehydes and reducing lipid peroxidation.

Metabolomic analysis showed that the exogenous ABA significantly altered the abundances of most lipid metabolites of tea leaves under drought stress. The abundances of LysoPE 14:0, LysoPE 16:0, LysoPE 18:0, LysoPE 18:1, LysoPC 15:1 and LysoPC 16:0 was significantly reduced and the abundances of PC 16:1/14:1, 13-HOTrE and MAG (18:3) isomer4 were slightly increased by exogenous ABA. To date, the association between the changes of these lipid metabolites and drought resistance of tea plants was rarely reported. While the correlation analysis of genes and metabolites showed that eight genes, *FATB*, *DGK1*, *EKI*, *ACAA1*, *GPX*, *DGK7*, *LPT1*, *EPHX2*, were significantly positive correlation with most lipid metabolites (including LysoPE 14:0, LysoPE 16:0, LysoPE 18:0, LysoPE 18:1, LysoPC 15:1 and LysoPC 16:0 ), and *LPCAT* and *ALDH* were significantly negative correlation with most lipid metabolites (Fig. 7, Supplementary Table S5), suggesting that the exogenous ABA could affect the expression of genes related to lipid metabolism for tea leaves to cause the reduction of lipid metabolites under drought stress. Previous study in *Vigna unguiculata* leaf demonstrated a role of ABA in the cell membrane protection against water stress by preventing drought-induced membrane lipid degradation^33^. Similar result was observed in the moss *A. androgynum* that the exogenous ABA might reduce the membrane damage by diminishing the lipid changes^22^. Previous research showed that the changes in lipid metabolism was correlated with alterations in membrane integrity under water stress^34,35^. In the present study of tea leaves, the results showed that exogenous ABA could mediate the expression of genes involved in lipid metabolism and affect the changes in lipid metabolism for maintaining membrane integrity and stability under drought stress. The interaction of ABA signaling with lipid signaling in tea plants under drought stress will be further elucidated in our future studies.

### Exogenous ABA increased the expression of genes related to flavonoid biosynthesis in tea leaves under drought stress

Flavonoids are ubiquitous secondary metabolites with a vast array of biological functions, including defense against biotic and abiotic stresses^36^. The flavonoid biosynthesis of tea plants was affected by various environmental conditions, such as drought stress^37^. In the study, we analyzed the effects of exogenous ABA on genes and metabolites related to flavonoid metabolism of tea leaves under drought stress using transcriptomic and metabonomic analysis.

Previous researches on tea plants showed that the expression of the genes related to flavonoid biosynthesis was firstly decreased and subsequently increased in response to drought stress, such as *CHS*, *DFR*, *LAR* and *ANR*, and the expression of *FLS* and *FNS* was continuously up-regulated in response to drought stress^3^. The expression of the genes related to flavonoid biosynthesis, such as *PAL*, *C4H*, *4CL*, *CHS* and *DFR*, was significantly induced by drought^1^. However, in tea plants, the effect of exogenous ABA on genes related to flavonoids biosynthetic in response to drought stress was rarely reported. In the present study, transcriptome analysis revealed that the exogenous ABA significantly induced the expression of genes related to flavonoid biosynthesis of tea leaves under drought stress. The expression of *PAL*, *4CL*, *F5′H* was highly down-regulated and the expression of *CHI*, *DRF*, *F3′H* and *FLS* was significantly up-regulated by exogenous ABA.

Several researches on tea plants showed that the amounts of total polyphenols and catechins, including GC, EGC, C, EC, EGCG, GCG, ECG, significantly decreased in response to drought stress^3,38–40^, but the content of total flavonoids significantly increased in response to drought stress^3^. However, in tea plants, there was limited information about the effect of exogenous ABA on flavonoid metabolites in response to drought stress. In the present study, metabolomics analysis revealed that the exogenous ABA significantly affected the abundances of flavonoid metabolites of tea leaves under drought stress. The abundances of flavone, anthocyanins, flavonol, isoflavone classes,including apigenin O-malonylhexoside, delphinidin 3-O-glucosidewere, cyanidin 3-O-rutinoside, kaempferitrin, sakuranetin, prunetin, kaempferol, fustin, ancyanidin 3,5-O-diglucoside, significantly increased and the abundances of flavonol, flavone and flavone C-glycosides, including dihydromyricetin, acacetin O-acetyl hexoside, acacetin O-glucuronic acid, 6-C-hexosyl-hesperetin O-hexoside, were slightly decreased by exogenous ABA.

We also performed the correlation analysis between the genes and flavonoid metabolites of tea leaves under exogenous ABA. The results showed that the expression of *CHI* (CSA026735) and *DFR* (CSA003949) was significantly positive correlation with apigenin O-malonylhexoside, delphinidin 3-O-glucoside, gallocatechin and dihydromyricetin, while the expression of *4CL* (CSA007753) was significantly negative correlation with apigenin O-malonylhexoside and delphinidin 3-O-glucoside, suggesting that the exogenous ABA could induce the expression of genes involved in flavonoid biosynthesis, and hence affected the biosynthesis of flavonoids and derivatives of tea leaves under drought stress.

Moreover, previous research showed that inhibition of lipid synthesis redirects the carbon flux into flavonoid metabolism^41^. Recently, *CHS*-mediated flavonoids were proven to repress embryonic fatty acid biosynthesis in the Arabidopsis seeds, implying a negative correlation between flavonoids and fatty acids biosynthesis in plants^42^. Our results showed that the exogenous ABA drastically decreased the abundances of lipid metabolites and increased the abundances of most flavonoids compared with severe drought. Therefore, another possible explanation was that the exogenous ABA induced the expression of genes involved in starch and sucrose metabolism to promote the accumulation of more carbon sources, and a large amount of carbon sources flux into flavonoid metabolism to increase the abundances of flavonoids and derivatives, so as to improve drought resistance of tea plants. Further studies are required to confirm such assumptions.

## Conclusion

In this study, we analyzed the roles of exogenous ABA on genes and metabolites of tea leaves under drought stress using transcriptomic and metabolomic analysis. The study demonstrated that exogenous ABA significantly reduce the damage of drought to tea plants, maintain the balance of primary metabolism, promote energy storages and the formation of flavonoids and derivatives to enhance drought tolerance of tea plants under drought stress. This study will be helpful for us to understand the mechanism of resilience to abiotic stress in tea plant and provide novel insights into enhancing drought tolerance of tea plants in the future.

## Material and methods

### Plant materials and stress treatments

Tea plant cultivar, ‘QN3’ (*Camellia sinensis* cv. QN3) is an improved cultivar bred by the Tea Research Institute, Qingdao Agricultural University^43–45^. The 2-year-old tea seedling were cultured in growth chamber for 2 weeks with photoperiod (12 h light at 25°C and 12 h dark at 20°C, 75% of humidity) and a light intensity of 18, 000 lx.

The experiment included four treatments: well-watered plants (CK); based on previous studies and physiological response of tea plants at different time points of drought stress^7^, the tea seedlings under drought stress for 24 h were defined as mild drought (MD), The tea seedlings of MD were divided into two parts: one was immediately sampled and the other was again divided into two groups: one group was immediately sprayed with 100 mL of ABA solution (ABA was purchased from Sigma Chemical Company, St. Louis, MO, USA), concentration 50 mg L^−1^. The other was immediately sprayed with equal parts of distilled water. Subsequently, the two groups were continuous drought with all of the other environmental conditions remaining constant. The leaves of two groups displayed visible morphological difference after another 53 h of drought (total 77 h of drought), the leaves of ABA treatment were sampled and defined as AT, another group was sampled and defined as severe drought (SD). Three independent biological replicates were performed at each treatment, and each replicate contained 10 mature leaves (the second fully expanded leaves at the top). All samples were frozen immediately in liquid nitrogen and stored at − 70°C.

### Measurement of physiological characterization

The contents of malondialdehyde (MDA) in the leaves were measured by the 2-thiobarbituric acid (TBA) method according to previous studies^12^. The leaf water content (LWC) of different treatment were determined as described previously^46^. The enzymatic assays of different treatment were determined as described previously^47^, including peroxidase (POD), catalase (CAT), ascorbate peroxidase (APX), glutathione reductase (GR). The contents of endogenous hormone of different treatment were determined as described previously^48^, including Ascorbic acid (ABA), indole-3-acetic acid (IAA) and Gibberellin (GA_3_). Leaf chlorophyll was extracted with ethanol according to previous studies^44^. Third leaf was sampled to measure maximum quantum yield of PSII according to previous studies^1^. All experiments were carried out with at least three independent repetitions.

### RNA-Seq analysis

For RNA-seq analysis, the total RNA of each sample was extracted as described by Wang et al.^49^. RNA concentration and integrity of the total RNA were measured using NanoDrop 2000 Spectrophotometer (IMPLEN, Westlake Village, CA, USA) and Agilent 2100 Bioanalyzer (Agilent Technologies, CA, USA), respectively. Subsequently, the library preparations were sequenced on the Illumina HiSeq platform to generate raw data. After sequencing, the clean reads were obtained by removing reads containing adaptors, more than 5% unknown bases and low-quality reads (> 20% of the bases with a quality score of ≤ 10). Gene function was annotated based on the following databases: NCBI non-redundant protein sequences (NR), Clusters of Orthologous (KOG/COG), Gene Ontology (GO), manually annotated and reviewed protein sequence database (Swiss-Prot), Kyoto Encyclopedia of Genes and Genomes (KEGG). Gene expression levels were represented using fragments per kilobase of transcript per million fragments mapped (FPKM) method. The differentially expressed genes (DEGs) were recruited based on False Discovery Rate (FDR) < 0.05 and |log_2_Fold Change|≥ 1. All DEGs were analyzed by GO enrichment using GOseq (1.10.0)^50^ and KEGG enrichment using KOBAS software^51,52^.

### Quantitative real-time RT-PCR (qRT-PCR) analysis

Total RNA was extracted by using a plant RNA extraction kit (Tiangen, Beijing, China). First-strand cDNA was reverse transcribed according to the user manual of PrimeScript RT reagent kit (TaKaRa), and the qRT-PCR program was performed by using a Light Cycler 480 instrument (Roche). The genes were selected for RT-qPCR with specific primers designed by Primer Premier 5 software (Supplementary Table S6). The qRT-PCR reagents were the following: a total of 20 μL of reaction mixture, which included 10 μL SYBR Green PCR Master Mix (Roche), 1 μL of each primer, 2 μL cDNA and 6 μL distilled water. The qPCR program was performed as follows: 95°C, 10 min; 95°C, 10 s and 60°C, 15 s for 45 cycles; then a melting curve. *GAPDH* (glyceraldehyde-3-phosphate dehydrogenase) was used as reference gene for quantifying the expression levels of the target genes according to the method of 2^−ΔΔCt^^53^. The quantitative analysis of each RNA sample was repeated at least three times, and the representative data are expressed as the mean values ± standard error (n = 3).

### Metabolite profiling analysis

Sample preparation, metabolite extraction and analysis were carried out as described in previous research^54,55^. In brief, the 100 mg freeze-dried samples were extracted using 1.0 mL 70% aqueous methanol (containing 0.1 mg/L lidocaine). Subsequently, 10,000*g* centrifugation for 10 min at 4 °C, then the extracts were absorbed and filtered before LC–MS analysis. A quality-control sample was prepared by equal blending of all samples; during the assay, the quality control sample was run every 10 injections to monitor the stability of the analytical conditions. The extracted samples (2 μL) were analyzed using a HPLC system (Shim-pack UFLC SHIMADZU CBM 30A) equipped with Waters ACQUITY UPLC HSS T3 C18 column (1.8 µm, 2.1 mm * 100 mm). LIT and triple quadrupole (QQQ) scans were acquired on a QTRAP-MS equipped with an ESI Turbo Ion-Spray interface under AB Sciex QTRAP 4,500 System, a positive ion mode and controlled by Analyst 1.6.1 software (AB Sciex). The solvent system, gradient program and ESI source operation parameters were carried out as described by previous research^56^.

The qualitative analysis of primary and secondary MS data was performed by searching the internal database using a self-compiled database MWDB (MetWare Biological Science and Technology Co., Ltd. Wuhan, China), Data pre-processing and metabolites identification were performed by the standard metabolic procedures, including comparing the m/z values, RT, and the fragmentation patterns with the standards. The variable importance of the projection (VIP) score of the application (O) PLS model was used to filter the best differentiated metabolites between treatments. Metabolites with significant differences in content were set with thresholds of variable importance in projection (VIP) ≥ 1 and fold change ≥ 2 or ≤ 0.5.

### Statistical analysis

All statistical analyses were performed with SPSS 20.0 (Windows; SPSS Inc., Chicago, IL, USA) and significance tests were determined by Duncan’s test and ANOVA. *P* < 0.05 was considered the significant difference. The relationships visualization was performed by Cytoscape software (version 3.3.0, USA). Integrative analysis of metabolome and transcriptome was carried out using R software (version 3.2.4, USA).

## Supplementary information

Supplementary legends.

Supplementary figure S1.

Supplementary figure S2.

Supplementary table S1.

Supplementary table S2.

Supplementary table S3.

Supplementary table S4.

Supplementary table S5.

Supplementary table S6.

## Data Availability

The datasets generated during the current study are available in the NCBI repository under the accession numbers PRJNA631326 after May. 2021.

## Abbreviations

ALDH: Aldehyde dehydrogenase
AT: ABA treatment
CAD: Cinnamyl-alcohol dehydrogenase
CCR: Cinnamoyl-CoA reductase
CHI3: Chalcone isomerase 3
CK: Well-watered plants
CHI: Chalcone isomerase
DEMs: Differential metabolites
DEGs: Differentially expressed genes
DFR: Dihydroflavonol 4-reductase
DGK: Diacylglycerol kinase
EKI: Ethanolamine kinase
FATB: Fatty acyl-ACP thioesterase B
FLS: Flavonol synthase
GO: Gene ontology
KEGG: Kyoto Encyclopedia of Genes and Genomes
MDA: Malondialdehyde
MD: Mild drought
PAL: Phenylalanine ammonia-lyase
PER42: Peroxidase 42
LPCAT: Lysophosphatidylcholine acyltransferase
SD: Severe drought
4CL: 4-Coumarate-CoA ligase
