# Supplementary material for: Exogenous abscisic acid induces the lipid and flavonoid metabolism of tea plants under drought stress
Source: Sci Rep. 2020 Jul 23;10:12275. doi: 10.1038/s41598-020-69080-1 (PMC7378251; doi:10.1038/s41598-020-69080-1)
Supplement: Supplementary file 1 — Supplementary legends. [file 41598_2020_69080_MOESM1_ESM.docx]

**Supplemental Figure S1.** GO enrichment analysis of DEGs. (A) GO enrichment analysis of DEGs in AT/MD. (B) GO enrichment analysis of DEGs in SD/MD. (C) GO enrichment analysis of DEGs in AT/SD. (D) GO enrichment analysis of DEGs in AT/CK. (E) GO enrichment analysis of DEGs in SD/CK. (F) GO enrichment analysis of DEGs in MD/AT. The figure was carried out using R software (version 3.2.4, USA).

**Supplemental Figure S2**. KEGG pathway analysis of DEGs. (A) KEGG pathway analysis of DEGs in AT/MD. (B) KEGG pathway analysis of DEGs in SD/MD. (C) KEGG pathway analysis of DEGs in AT/SD. (D) KEGG pathway analysis of DEGs in AT/CK. (E) KEGG pathway analysis of DEGs in SD/CK. (F) KEGG pathway analysis of DEGs in MD/AT. The figure was carried out using R software (version 3.2.4, USA).

**Supplemental Table S1**. Throughput and quality summary of RNA-sequence.

**Supplemental Table S2**. The genes related to energy metabolism and amino acid metabolism.

**Supplemental Table S3.** The fold changes of differential metabolites in lipid metabolism.

**Supplemental Table S4**. The connection analysis between the genes and metabolites related to flavonoid.

**Supplemental Table S5.** The connection analysis between the genes and metabolites related to lipid metabolism.

**Supplemental Table S6**. Primer used for qRT-PCR.
