# Supplementary material for: Exogenous abscisic acid induces the lipid and flavonoid metabolism of tea plants under drought stress
Source: Sci Rep. 2020 Jul 23;10:12275. doi: 10.1038/s41598-020-69080-1 (PMC7378251; doi:10.1038/s41598-020-69080-1)
Supplement: Supplementary file 3 — Supplementary figure S2. [file 41598_2020_69080_MOESM3_ESM.pdf]

Exogenous abscisic acid induces the lipid and flavonoid metabolism of tea plants under drought stress

Zhongshuai Gai [12#], Yu Wang[1#], Yiqian Ding[1], Wenjun Qian[1], Chen Qiu[1], Hui Xie[1], Litao Sun[1], Zhongwu Jiang[2], Qingping Ma[3], Linjun Wang[4], Zhaotang Ding[1]*

[1]Tea Research Institute, Qingdao Agricultural University, Qingdao 266109, China

[2]College of Life Science, Yantai University, Yantai, Shandong, 264005, China

[3]College of agriculture, Liaocheng University, Liaocheng, Shandong, 252059, China

[4]Fruit tea station of weihai agricultural and rural affairs service center, Weihai, Shandong, 264200, China

# These authors contributed equally to this study.

***CORRESPONDENCE:**

Dr Zhaotang Ding

E-mails: dzttea@163.com

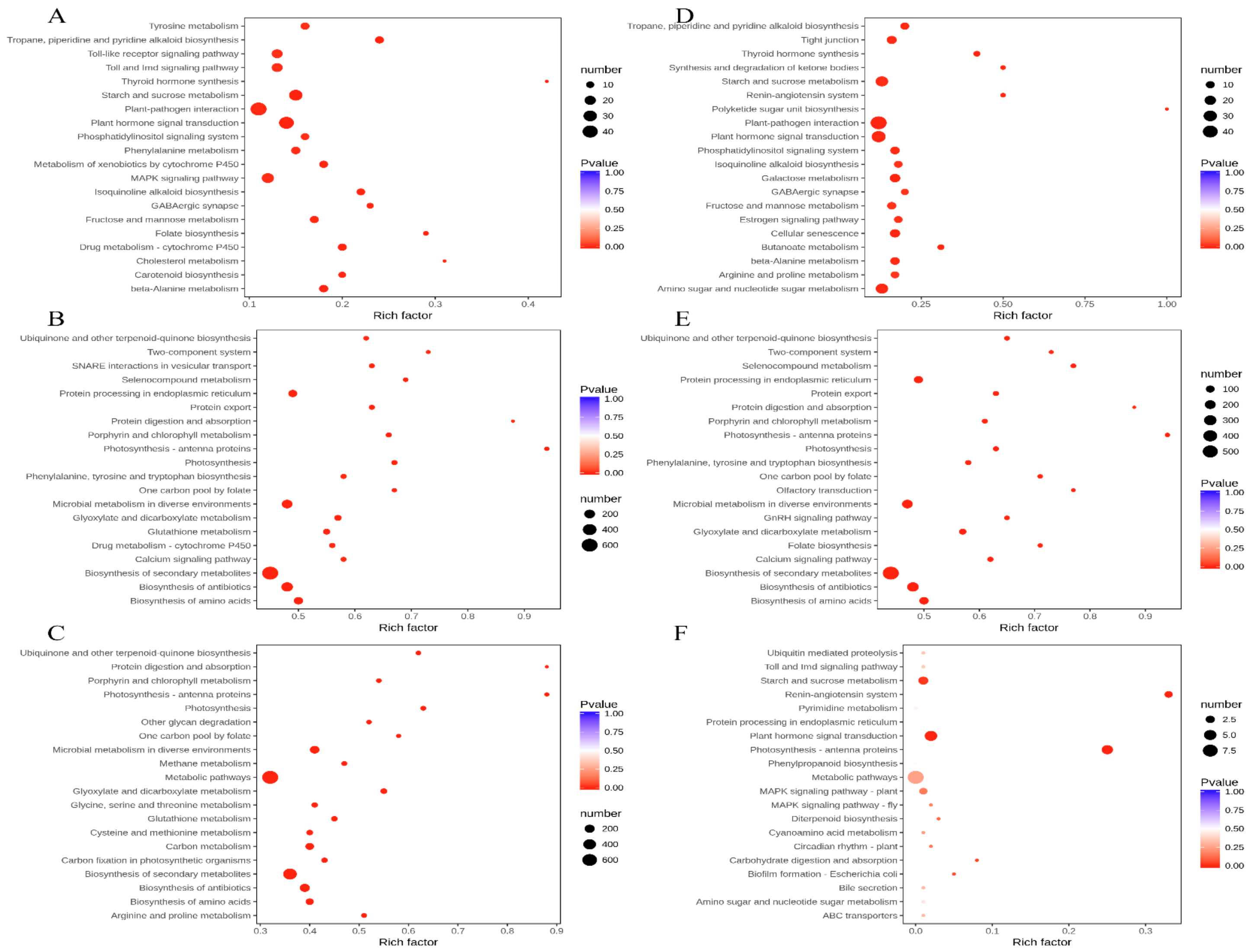

Supplemental Figure S2. KEGG pathway analysis of DEGs. (A) KEGG pathway analysis of DEGs in AT/MD. (B) KEGG pathway analysis of DEGs in SD/MD. (C) KEGG pathway analysis of DEGs in AT/SD. The figure was carried out using R software (version 3.2.4, USA).
