# Supplementary material for: Exogenous abscisic acid induces the lipid and flavonoid metabolism of tea plants under drought stress
Source: Sci Rep. 2020 Jul 23;10:12275. doi: 10.1038/s41598-020-69080-1 (PMC7378251; doi:10.1038/s41598-020-69080-1)
Supplement: Supplementary file 4 — Supplementary table S1. [file 41598_2020_69080_MOESM4_ESM.pdf]

Exogenous abscisic acid induces the lipid and flavonoid metabolism of tea plants under drought stress

Zhongshuai Gai [12#], Yu Wang[1#], Yiqian Ding[1], Wenjun Qian[1], Chen Qiu[1], Hui Xie[1], Litao Sun[1], Zhongwu Jiang[2], Qingping Ma[3], Linjun Wang[4], Zhaotang Ding[1]*
[1]Tea Research Institute, Qingdao Agricultural University, Qingdao 266109, China
[2]College of Life Science, Yantai University, Yantai, Shandong, 264005, China
[3]College of agriculture, Liaocheng University, Liaocheng, Shandong, 252059, China
[4]Fruit tea station of weihai agricultural and rural affairs service center, Weihai, Shandong, 264200, China
# These authors contributed equally to this study.
***CORRESPONDENCE:** Dr Zhaotang Ding E-mails: dzttea@163.com

Table S1 Quality of sequencing

Samples	Clean reads	Cleans bases（G）	Error（%）	Q20（%）	Q30（%）	GC(%）
CK1	53,324,234	8.0	0.34	97.29	92.97	44.52
CK2	50,912,316	7.64	0.36	97.09	92.54	44.50
CK3	49,215,718	7.38	0.33	97.37	93.22	44.70
MD1	47,871,864	7.18	0.33	97.34	93.18	45.06
MD2	53,180,726	7.98	0.32	97.48	93.44	44.80
MD3	52,383,110	7.86	0.33	97.38	93.34	44.98
AT1	51,863,816	7.78	0.33	97.35	93.29	45.14
AT2	53,845,492	8.08	0.33	97.37	93.41	45.61
AT3	52,340,128	7.85	0.32	97.48	93.66	45.66
SD1	51,611,966	7.74	0.33	97.35	93.20	44.89
SD2	51,740,376	7.76	0.33	97.40	93.36	45.01
SD3	51,528,212	7.73	0.34	97.27	93.10	44.57

CK: control group; MD: mild drought group; AT: ABA treatment group; SD: severe drought group
