# Supplementary material for: Exogenous abscisic acid induces the lipid and flavonoid metabolism of tea plants under drought stress
Source: Sci Rep. 2020 Jul 23;10:12275. doi: 10.1038/s41598-020-69080-1 (PMC7378251; doi:10.1038/s41598-020-69080-1)
Supplement: Supplementary file 5 — Supplementary table S2. [file 41598_2020_69080_MOESM5_ESM.pdf]

Exogenous abscisic acid induces the lipid and flavonoid metabolism of tea plants under drought stress

Zhongshuai Gai 12#, Yu Wang1#, Yiqian Ding1, Wenjun Qian1, Chen Qiu1, Hui Xie1, Litao Sun1, Zhongwu Jiang2, Qingping Ma3, Linjun Wang4, Zhaotang Ding1*

[1]Tea Research Institute, Qingdao Agricultural University, Qingdao 266109, China

[2]College of Life Science, Yantai University, Yantai, Shandong, 264005, China

[3]College of agriculture, Liaocheng University, Liaocheng, Shandong, 252059, China

[4]Fruit tea station of weihai agricultural and rural affairs service center, Weihai, Shandong, 264200, China

# These authors contributed equally to this study.

*CORRESPONDENCE: Zhaotang Ding, E-mails: dzttea@163.com

	Gene ID	KEGG
	CSA006152	K00844: HK; hexokinase [EC:2.7.1.1]
HK	CSA018986	K00844: HK; hexokinase [EC:2.7.1.1]
	CSA020560	K00844: HK; hexokinase [EC:2.7.1.1]
G6PI	CSA017525	K01810: GPI, pgi; glucose-6-phosphate isomerase [EC:5.3.1.9]
	CSA015866	K00850: pfkA, PFK; 6-phosphofructokinase 1 [EC:2.7.1.11]
PFK	CSA020365	K00850: pfkA, PFK; 6-phosphofructokinase 1 [EC:2.7.1.11]
	CSA030565	K00850: pfkA, PFK; 6-phosphofructokinase 1 [EC:2.7.1.11]
	CSA032544	K00850: pfkA, PFK; 6-phosphofructokinase 1 [EC:2.7.1.11]
	CSA005116	K01623: ALDO; fructose-bisphosphate aldolase, class I [EC:4.1.2.13]
	CSA009966	K01623: ALDO; fructose-bisphosphate aldolase, class I [EC:4.1.2.13]
Aldolase	CSA011908	K01623: ALDO; fructose-bisphosphate aldolase, class I [EC:4.1.2.13]
	CSA035207	K01623: ALDO; fructose-bisphosphate aldolase, class I [EC:4.1.2.13]
GAPDH	CSA023938	K00134: GAPDH, gapA; glyceraldehyde 3-phosphate dehydrogenase [EC:1.2.1.12]
PGK	CSA013128	PGKH,RecName: Full=Phosphoglycerate kinase; EC=2.7.2.3
	CSA013129	PGKH,RecName: Full=Phosphoglycerate kinase, chloroplastic; EC=2.7.2.3
PGAM	CSA017497	K01834: PGAM; 2,3-bisphosphoglycerate-dependent phosphoglycerate mutase [EC:5.4.
ENO	CSA007194	K01689: ENO, eno; enolase [EC:4.2.1.11]
	CSA019642	K00873: PK, pyk; pyruvate kinase [EC:2.7.1.40]
	CSA025782	K00873: PK, pyk; pyruvate kinase [EC:2.7.1.40]
PK	CSA025784	K00873: PK, pyk; pyruvate kinase [EC:2.7.1.40]
	CSA032877	K00873: PK, pyk; pyruvate kinase [EC:2.7.1.40]
	CSA003073	K00382: DLD, lpd, pdhD; dihydrolipoamide dehydrogenase [EC:1.8.1.4]
	CSA035439	K00382: DLD, lpd, pdhD; dihydrolipoamide dehydrogenase [EC:1.8.1.4]
PDC	CSA005663	K00627: DLAT,pyruvate dehydrogenase E2 component [EC:2.3.1.12]
	CSA033762	K00627: DLAT, pyruvate dehydrogenase E2 component [EC:2.3.1.12]
	CSA016421	K01648: ACLY; ATP citrate (pro-S)-lyase [EC:2.3.3.8]
	CSA020419	K01648: ACLY; ATP citrate (pro-S)-lyase [EC:2.3.3.8]
	CSA027464	K01648: ACLY; ATP citrate (pro-S)-lyase [EC:2.3.3.8]
ACLY	CSA029711	K01648: ACLY; ATP citrate (pro-S)-lyase [EC:2.3.3.8]
	CSA033095	K01648: ACLY; ATP citrate (pro-S)-lyase [EC:2.3.3.8]
	CSA033096	K01648: ACLY; ATP citrate (pro-S)-lyase [EC:2.3.3.8]
	CSA034373	K01648: ACLY; ATP citrate (pro-S)-lyase [EC:2.3.3.8]
CS	CSA009600	K01647: CS, gltA; citrate synthase [EC:2.3.3.1]
	CSA017504	K01647: CS, gltA; citrate synthase [EC:2.3.3.1]
	CSA024497	K01681: ACO, acnA; aconitate hydratase [EC:4.2.1.3]
Aconitase	CSA024499	K01681: ACO, acnA; aconitate hydratase [EC:4.2.1.3]
	CSA025704	K01681: ACO, acnA; aconitate hydratase [EC:4.2.1.3]
	CSA011175	K00030: IDH3; isocitrate dehydrogenase (NAD+) [EC:1.1.1.41]
IDH	CSA016077	K00030: IDH3; isocitrate dehydrogenase (NAD+) [EC:1.1.1.41]
	CSA016078	K00030: IDH3; isocitrate dehydrogenase (NAD+) [EC:1.1.1.41]
PDHE	CSA032487	K00162: PDHB, pdhB; pyruvate dehydrogenase E1 component beta subunit [EC:1.2.4.1
OGD	CSA019997	K00658: DLST, sucB; 2-oxoglutarate dehydrogenase E2 component [EC:2.3.1.61]
	CSA028417	K00658: DLST, sucB; 2-oxoglutarate dehydrogenase E2 component[EC:2.3.1.61]
DLD	CSA003073	K00382: DLD, lpd, pdhD; dihydrolipoamide dehydrogenase [EC:1.8.1.4]
	CSA035439	K00382: DLD, lpd, pdhD; dihydrolipoamide dehydrogenase [EC:1.8.1.4]
SCS	CSA028548	K01899: LSC1; succinyl-CoA synthetase alpha subunit [EC:6.2.1.4 6.2.1.5]
	CSA002464	SDHA1: Full=Succinate dehydrogenase [ubiquinone] flavoprotein subunit 1; EC=1.3.5.1
SDH	CSA021044	SDH7B: Full=Succinate dehydrogenase subunit 7B, mitochondrial {ECO:0000305}
	CSA035376	SDAF2 : Full=Succinate dehydrogenase {ECO:0000305}; Short=SDH
FUM	CSA004162	K01679: fumC; fumarate hydratase, class II [EC:4.2.1.2]
	CSA030699	K00025: MDH1; malate dehydrogenase [EC:1.1.1.37]
MDH		

MDH	CSA009850 K00026: MDH2; malate dehydrogenase [EC:1.1.1.37]
PYG	CSA002692 K00688: PYG, glgP; glycogen phosphorylase [EC:2.4.1.1]
	CSA013684 K00688: PYG, glgP; glycogen phosphorylase [EC:2.4.1.1]
UGP	CSA013531 K00963: UGP2; UTP--glucose-1-phosphate uridylyltransferase [EC:2.7.7.9]
SUS	CSA034422 K00695: E2.4.1.13; sucrose synthase [EC:2.4.1.13]
α-glucosidas	CSA008742 K01187: malZ; alpha-glucosidase [EC:3.2.1.20]
Invertase	CSA016742 K01193: INV, sacA; beta-fructofuranosidase [EC:3.2.1.26]
	CSA020598 K01193: INV, sacA; beta-fructofuranosidase [EC:3.2.1.26]
	CSA021680 K01193: INV, sacA; beta-fructofuranosidase [EC:3.2.1.26]
	CSA022665 K01193: INV, sacA; beta-fructofuranosidase [EC:3.2.1.26]
	CSA030104 K01193: INV, sacA; beta-fructofuranosidase [EC:3.2.1.26]
AMY	CSA030401 K01176: AMY, amyA, malS; alpha-amylase [EC:3.2.1.1]
	CSA033527 K01176: AMY, amyA, malS; alpha-amylase [EC:3.2.1.1]
BAM	CSA002350 K01177: E3.2.1.2; beta-amylase [EC:3.2.1.2]
	CSA003843 K01177: E3.2.1.2; beta-amylase [EC:3.2.1.2]
	CSA010521 K01177: E3.2.1.2; beta-amylase [EC:3.2.1.2]
	CSA011693 K01177: E3.2.1.2; beta-amylase [EC:3.2.1.2]
	CSA020619 K01177: E3.2.1.2; beta-amylase [EC:3.2.1.2]
	CSA033012 K01177: E3.2.1.2; beta-amylase [EC:3.2.1.2]
GAD	CSA004789 K01580: GAD; glutamate decarboxylase [EC:4.1.1.15]
	CSA004790 K01580: GAD; glutamate decarboxylase [EC:4.1.1.15]
	CSA025312 K01580: GAD; glutamate decarboxylase [EC:4.1.1.15]
GS	CSA001114 K01915: glnA, GLUL; glutamine synthetase [EC:6.3.1.2]
	CSA006044 K01915: glnA, GLUL; glutamine synthetase [EC:6.3.1.2]
	CSA009334 K01915: glnA, GLUL; glutamine synthetase [EC:6.3.1.2]
	CSA010785 K01915: glnA, GLUL; glutamine synthetase [EC:6.3.1.2]
AAT	CSA001188 K14455: GOT2; aspartate aminotransferase, mitochondrial [EC:2.6.1.1]
ASNS	CSA000266 K01953: ASNS; asparagine synthase (glutamine-hydrolysing) [EC:6.3.5.4]
	CSA035384 K01953: ASNS; asparagine synthase (glutamine-hydrolysing) [EC:6.3.5.4]
GOGAT	CSA027515 K00264: GLT1; glutamate synthase (NADPH/NADH) [EC:1.4.1.13 1.4.1.14]
SHMT	CSA004046 K00600: glyA, SHMT; glycine hydroxymethyltransferase [EC:2.1.2.1]
	CSA009632 K00600: glyA, SHMT; glycine hydroxymethyltransferase [EC:2.1.2.1]
	CSA010617 K00600: glyA, SHMT; glycine hydroxymethyltransferase [EC:2.1.2.1]
	CSA021546 K00600: glyA, SHMT; glycine hydroxymethyltransferase [EC:2.1.2.1]
	CSA022821 K00600: glyA, SHMT; glycine hydroxymethyltransferase [EC:2.1.2.1]
	CSA025613 K00600: glyA, SHMT; glycine hydroxymethyltransferase [EC:2.1.2.1]
	CSA026298 K00600: glyA, SHMT; glycine hydroxymethyltransferase [EC:2.1.2.1]
PSAT	CSA005241 K00831: serC, PSAT1; phosphoserine aminotransferase [EC:2.6.1.52]
TAT	CSA010886 K00815: TAT; tyrosine aminotransferase [EC:2.6.1.5]
	CSA021496 K00815: TAT; tyrosine aminotransferase [EC:2.6.1.5]
	CSA025650 K00815: TAT; tyrosine aminotransferase [EC:2.6.1.5]
ADT/PDT	CSA005061 K05359: ADT, PDT; arogenate/prephenate dehydratase [EC:4.2.1.91 4.2.1.51]
	CSA015842 K05359: ADT, PDT; arogenate/prephenate dehydratase [EC:4.2.1.91 4.2.1.51]
	CSA031339 K05359: ADT, PDT; arogenate/prephenate dehydratase [EC:4.2.1.91 4.2.1.51]
ADH	CSA006042 K15227: TYRAAT; arogenate dehydrogenase (NADP+), plant [EC:1.3.1.78]
HMT	CSA000372 K00547: mmuM, BHMT2; homocysteine S-methyltransferase [EC:2.1.1.10]
	CSA024522 K00547: mmuM, BHMT2; homocysteine S-methyltransferase [EC:2.1.1.10]
METE	CSA020814 K00549: metE; 5-methyltetrahydropteroyltriglutamate- [EC:2.1.1.14]
	CSA026696 K00549: metE; 5-methyltetrahydropteroyltriglutamate- [EC:2.1.1.14]
	CSA034466 K00549: metE; 5-methyltetrahydropteroyltriglutamate- [EC:2.1.1.14]
	CSA036570 K00549: metE; 5-methyltetrahydropteroyltriglutamate- [EC:2.1.1.14]
CYSK	CSA000873 K01738: cysK; cysteine synthase A [EC:2.5.1.47]
	CSA007571 K01738: cysK; cysteine synthase A [EC:2.5.1.47]
ILVC	CSA003623 K00053: ilvC; ketol-acid reductoisomerase [EC:1.1.1.86]
ALT	CSA024958 K00826: ilvE; branched-chain amino acid aminotransferase [EC:2.6.1.42]
ILVD	CSA012062 K01687: ilvD; dihydroxy-acid dehydratase [EC:4.2.1.9]
