# Supplementary material for: Exogenous abscisic acid induces the lipid and flavonoid metabolism of tea plants under drought stress
Source: Sci Rep. 2020 Jul 23;10:12275. doi: 10.1038/s41598-020-69080-1 (PMC7378251; doi:10.1038/s41598-020-69080-1)
Supplement: Supplementary file 6 — Supplementary table S3. [file 41598_2020_69080_MOESM6_ESM.pdf]

Table S3 The fold changes of differential metabolites in lipid metabolism

Class	Compounds	Log2FC （AT/MD）	Log2FC (SD/MD)	Log2FC (AT/SD)
**Glycerophospholipids**	LysoPC 16:1	**1.37**	**2.79**	**-1.42**
	PC 16:1/14:1	**1.13**	0.94	0.19
	LysoPC 18:2	**1.39**	**2.70**	**-1.31**
	LysoPC 18:3	**1.76**	**2.85**	**-1.09**
	LysoPC 16:0	**2.13**	**3.19**	**-1.05**
	LysoPE 14:0	**2.07**	**3.40**	**-1.33**
	LysoPC 14:0	**1.68**	**2.79**	**-1.11**
	LysoPE 18:2 (2n isomer)	**1.70**	**3.30**	**-1.60**
	LysoPE 16:0	**2.55**	**4.01**	**-1.46**
	LysoPE 18:2	**-1.50**	**-1.23**	-0.27
	LysoPC 18:1	**1.28**	**2.95**	**-1.67**
	LysoPE 18:0	**2.25**	**3.88**	**-1.63**
	LysoPC 15:1	**1.40**	**3.16**	**-1.77**
	LysoPC 15:0	**1.84**	**2.85**	**-1.01**
	LysoPC 17:0	**2.41**	**3.33**	-0.93
	LysoPE 18:1	**1.92**	**3.68**	**-1.76**
	LysoPC 16:0 (2n isomer)	**1.91**	**2.41**	-0.50
	LysoPS 22:6	**10.00**	**11.57**	**-1.56**
	LysoPC 16:1 (2n isomer)	0.63	**1.91**	**-1.28**
	LysoPC 18:1 (2n isomer)	0.78	**1.94**	**-1.16**
	LysoPC 16:2 (2n isomer)	0.98	**1.94**	-0.95
	LysoPC 18:3 (2n isomer)	0.49	**1.24**	-0.75
	LysoPC 18:2 (2n isomer)	0.60	**1.97**	**-1.37**
	LysoPC 18:0 (2n isomer)	0.84	**2.10**	**-1.26**
	LysoPE 18:1 (2n isomer)	0.11	**1.30**	**-1.19**
	LysoPC 20:4	0.43	**1.81**	**-1.38**
**Glycerolipids**	MAG (18:4) isomer3	**1.20**	**1.76**	-0.55
	MAG (18:3) isomer3	**-1.28**	-0.92	-0.36
	MAG (18:4) isomer1	0.52	**1.09**	-0.57
	MAG (18:1) isomer2	0.07	**1.42**	**-1.36**
	MAG (18:3) isomer4	-0.62	**-1.12**	0.50
	MAG (18:1) isomer1	0.14	**1.54**	**-1.40**
	DGMG (18:2) isomer1	-0.13	0.96	**-1.10**
	DGMG (18:2) isomer3	-0.19	0.98	**-1.17**
**Fatty acids**	13-HOTrE	**1.03**	0.97	0.06
	13-HPODE	**1.00**	**1.26**	-0.26
	"9-Hydroxy-(10E,12Z,15Z)-octadecatrienoic acid"	0.91	**1.45**	-0.54
	"4-oxo-9Z,11Z,13E,15E-octadecatetraenoic acid"	0.86	**1.34**	-0.47

Note: The FCs were described with the log2 transformed numbers, the bold fonts indicate significant difference metabolites in AT/MD, SD/MD and AT/SD. The positive number indicates the increase and negative numbers indicates the decrease, MD: mild drought group; AT: ABA treatment group; SD: severe drought group.
