# Supplementary material for: Exogenous abscisic acid induces the lipid and flavonoid metabolism of tea plants under drought stress
Source: Sci Rep. 2020 Jul 23;10:12275. doi: 10.1038/s41598-020-69080-1 (PMC7378251; doi:10.1038/s41598-020-69080-1)
Supplement: Supplementary file 7 — Supplementary table S4. [file 41598_2020_69080_MOESM7_ESM.pdf]

Exogenous abscisic acid induces the lipid and flavonoid metabolism of tea plants under drought stress

Zhongshuai Gai 12#, Yu Wang1#, Yiqian Ding1, Wenjun Qian1, Chen Qiu1, Hui Xie1, Litao Sun1, Zhongwu Jiang2, Qingping Ma3, Linjun Wang4, Zhaotang Ding1*
1Tea Research Institute, Qingdao Agricultural University, Qingdao 266109, China
2College of Life Science, Yantai University, Yantai, Shandong, 264005, China
3College of agriculture, Liaocheng University, Liaocheng, Shandong, 252059, China
4Fruit tea station of weihai agricultural and rural affairs service center, Weihai, Shandong, 264200, China
# These authors contributed equally to this study.
*CORRESPONDENCE: Zhaotang Ding, E-mails: dzttea@163.com

Gene ID	KEGG
CSA022970	K05349: beta-glucosidase [EC:3.2.1.21]
CSA022832	K22395:cinnamyl-alcohol dehydrogenase [EC:1.1.1.195]
CSA026155	K00083: cinnamyl-alcohol dehydrogenase [EC:1.1.1.195]
CSA001158	K00487: trans-cinnamate 4-monooxygenase [EC:1.14.13.11]
CSA024718	K00660: CHS; chalcone synthase [EC:2.3.1.74]
CSA026735	K01859: chalcone isomerase [EC:5.5.1.6]
CSA023140	K13065: HCT; shikimate O-hydroxycinnamoyltransferase [EC:2.3.1.133]
CSA033933	K13065: HCT; shikimate O-hydroxycinnamoyltransferase [EC:2.3.1.133]
CSA005167	K00430: peroxidase [EC:1.11.1.7]
CSA008527	K00430: peroxidase [EC:1.11.1.7]
CSA032145	K01188: beta-glucosidase [EC:3.2.1.21]
CSA022024	K10775: PAL; phenylalanine ammonia-lyase [EC:4.3.1.24]
CSA016635	K12355: REF1; coniferyl-aldehyde dehydrogenase [EC:1.2.1.68]
CSA021736	K00430: peroxidase [EC:1.11.1.7]
CSA032093	K00430: peroxidase [EC:1.11.1.7]
CSA034169	K00588: caffeoyl-CoA O-methyltransferase [EC:2.1.1.104]
CSA028450	K10775: PAL; phenylalanine ammonia-lyase [EC:4.3.1.24]
CSA004930	K00475: naringenin 3-dioxygenase [EC:1.14.11.9]
CSA033689	K01859: chalcone isomerase [EC:5.5.1.6]
CSA003949	K13082: DFR; flavanone 4-reductase [EC:1.1.1.219 1.1.1.234]
CSA003315	K00430: peroxidase [EC:1.11.1.7]
CSA027568	K00430: peroxidase [EC:1.11.1.7]
CSA030594	K01188: beta-glucosidase [EC:3.2.1.21]
CSA007753	K01904: 4CL; 4-coumarate--CoA ligase [EC:6.2.1.12]
CSA004504	K05350: beta-glucosidase [EC:3.2.1.21]
CSA000931	K09753: CCR; cinnamoyl-CoA reductase [EC:1.2.1.44]
CSA007350	K09753: CCR; cinnamoyl-CoA reductase [EC:1.2.1.44]
CSA007894	K13065: HCT; shikimate O-hydroxycinnamoyltransferase [EC:2.3.1.133]
CSA032390	K00083： cinnamyl-alcohol dehydrogenase [EC:1.1.1.195]
CSA033806	K01188: beta-glucosidase [EC:3.2.1.21]
CSA014862	K00430: peroxidase [EC:1.11.1.7]
CSA008201	K09755: F5H; ferulate-5-hydroxylase
CSA002127	K13082: DFR; flavanone 4-reductase [EC:1.1.1.219 1.1.1.234]
CSA020376	K00083: cinnamyl-alcohol dehydrogenase [EC:1.1.1.195]
CSA032745	K00083: cinnamyl-alcohol dehydrogenase [EC:1.1.1.195]
CSA006767	K00430: peroxidase [EC:1.11.1.7]
CSA018157	K00430: peroxidase [EC:1.11.1.7]
CSA018401	K00430: peroxidase [EC:1.11.1.7]
CSA028049	K00430: peroxidase [EC:1.11.1.7]
CSA032184	K00430: peroxidase [EC:1.11.1.7]
CSA032034	K01188: beta-glucosidase [EC:3.2.1.21]
CSA026844	K05349:beta-glucosidase [EC:3.2.1.21]
CSA006900	K09753: cinnamoyl-CoA reductase [EC:1.2.1.44]

Index	Compounds
pmb0621	C-hexosyl-isorhamnetin O-hexoside
pmb2987	Acacetin O-acetyl hexoside
pme2895	Dihydromyricetin
pme3407	Laricitrin
pmb0576	Apigenin O-malonylhexoside
pmb0645	6-C-hexosyl-hesperetin O-hexoside
pmb0672	6-C-hexosyl-apigenin O-feruloylhexoside
pmb0835	Gallocatechin-gallocatechin
pme1398	Delphinidin 3-O-glucoside (Mirtillin)
pme1773	Cyanidin 3-O-rutinoside (Keracyanin)
pme2491	"Kaempferol 3,7-dirhamnoside (Kaempferitrin)"
pme3396	Fustin

geneName	metaName	R^2

CSA022970	pmb0621	0.833
CSA022832	pmb0621	0.839
CSA026155	pmb2987	-0.832
CSA001158	pme2895	-0.864
CSA024718	pme2895	0.999
CSA026735	pme2895	0.933
CSA023140	pme2895	0.908
CSA033933	pme2895	0.837
CSA005167	pme2895	-0.988
CSA008527	pme2895	-0.984
CSA032145	pme2895	-0.987
CSA022024	pme2895	-0.82
CSA016635	pme2895	-0.929
CSA021736	pme2895	0.958
CSA032093	pme2895	0.903
CSA034169	pme3407	-0.832
CSA028450	pme3407	-0.812
CSA004930	pmb0576	0.89
CSA026735	pmb0576	0.852
CSA033689	pmb0576	-0.865
CSA003949	pmb0576	0.854
CSA003315	pmb0576	0.85
CSA027568	pmb0576	0.806
CSA030594	pmb0576	-0.803
CSA007753	pmb0576	-0.805
CSA004504	pmb0576	0.955
CSA000931	pmb0576	0.919
CSA007350	pmb0576	0.904
CSA016635	pmb0576	-0.86
CSA032093	pmb0576	0.899
CSA007894	pmb0645	0.814
CSA032390	pmb0645	-0.811
CSA003315	pmb0645	-0.804
CSA033806	pmb0672	0.891
CSA001158	pmb0835	-0.801
CSA024718	pmb0835	0.813
CSA026735	pmb0835	0.887
CSA003949	pmb0835	0.829
CSA008527	pmb0835	-0.857
CSA014862	pmb0835	-0.823
CSA000931	pmb0835	0.812
CSA016635	pmb0835	-0.852
CSA032093	pmb0835	0.846
CSA004930	pme1398	0.888
CSA026735	pme1398	0.852
CSA033689	pme1398	-0.863
CSA003949	pme1398	0.851
CSA003315	pme1398	0.847
CSA027568	pme1398	0.805
CSA030594	pme1398	-0.802
CSA007753	pme1398	-0.803
CSA004504	pme1398	0.953
CSA000931	pme1398	0.917
CSA007350	pme1398	0.901
CSA016635	pme1398	-0.861
CSA032093	pme1398	0.899
CSA014862	pme1773	-0.871
CSA008201	pme2491	0.851
CSA002127	pme3396	0.973
CSA020376	pme3396	-0.946
CSA032390	pme3396	0.807
CSA032745	pme3396	-0.863
CSA006767	pme3396	0.923
CSA018157	pme3396	0.806
CSA018401	pme3396	0.959
CSA028049	pme3396	-0.839
CSA032184	pme3396	-1
CSA030594	pme3396	-0.82
CSA032034	pme3396	-0.999
CSA026844	pme3396	-0.847
CSA006900	pme3396	0.969
