# Supplementary material for: Exogenous abscisic acid induces the lipid and flavonoid metabolism of tea plants under drought stress
Source: Sci Rep. 2020 Jul 23;10:12275. doi: 10.1038/s41598-020-69080-1 (PMC7378251; doi:10.1038/s41598-020-69080-1)
Supplement: Supplementary file 8 — Supplementary table S5. [file 41598_2020_69080_MOESM8_ESM.pdf]

Exogenous abscisic acid induces the lipid and flavonoid metabolism of tea plants under drought stress

Zhongshuai Gai 12#, Yu Wang1#, Yiqian Ding1, Wenjun Qian1, Chen Qiu1, Hui Xie1, Litao Sun1, Zhongwu Jiang2, Qingping Ma3, Linjun Wang4, Zhaotang Ding1*

[1]Tea Research Institute, Qingdao Agricultural University, Qingdao 266109, China

[2]College of Life Science, Yantai University, Yantai, Shandong, 264005, China

[3]College of agriculture, Liaocheng University, Liaocheng, Shandong, 252059, China

[4]Fruit tea station of weihai agricultural and rural affairs service center, Weihai, Shandong, 264200, China

# These authors contributed equally to this study.

*CORRESPONDENCE: Zhaotang Ding, E-mails: dzttea@163.com

Gene ID	KEGG
CSA035088	K08726: EPHX2; lipid-phosphate phosphatase [EC:3.3.2.10 3.1.3.76]
CSA031747	K00901: DGK; diacylglycerol kinase (ATP) [EC:2.7.1.107]
CSA004606	K03715: MGD; 1,2-diacylglycerol 3-beta-galactosyltransferase [EC:2.4.1.46]
CSA030599	K00894: ETNK, EKI; ethanolamine kinase [EC:2.7.1.82]
CSA014346	K00432: gpx; glutathione peroxidase [EC:1.11.1.9]
CSA011597	K08726: EPHX2; lipid-phosphate phosphatase [EC:3.3.2.10 3.1.3.76]
CSA012072	K00967: PCYT2; ethanolamine-phosphate cytidylyltransferase [EC:2.7.7.14]
CSA034138	K01094: GEP4; phosphatidylglycerophosphatase GEP4 [EC:3.1.3.27]
CSA033891	K01115: PLD1_2; phospholipase D1/2 [EC:3.1.4.4]
CSA016252	K01126: glpQ, ugpQ; glycerophosphoryl diester phosphodiesterase [EC:3.1.4.46]
CSA018764	K01613: psd, PISD; phosphatidylserine decarboxylase [EC:4.1.1.65]
CSA001056	K13510: LPCAT1_2; lysophosphatidylcholine acyltransferase [EC:2.3.1.23 2.3.1.67]
CSA019962	K15718: LOX1_5; linoleate 9S-lipoxygenase [EC:1.13.11.58]
CSA005325	K00232: ACOX1, ACOX3; acyl-CoA oxidase [EC:1.3.3.6]
CSA012667	K00454: LOX2S; lipoxygenase [EC:1.13.11.12]
CSA016222	K00454: LOX2S; lipoxygenase [EC:1.13.11.12]
CSA000654	K05894: OPR; 12-oxophytodienoic acid reductase [EC:1.3.1.42]
CSA001069	K07513: ACAA1; acetyl-CoA acyltransferase 1 [EC:2.3.1.16]
CSA013647	K10526: OPCL1; OPC-8:0 CoA ligase 1 [EC:6.2.1.-]
CSA000775	K18857: ADH1; alcohol dehydrogenase class-P [EC:1.1.1.1]
CSA005266	K00002: AKR1A1, adh; alcohol dehydrogenase (NADP+) [EC:1.1.1.2]
CSA005242	K00128: ALDH; aldehyde dehydrogenase (NAD+) [EC:1.2.1.3]
CSA031091	K00901: DGK; diacylglycerol kinase (ATP) [EC:2.7.1.107]
CSA032782	K13519: LPT1, ALE1; lysophospholipid acyltransferase [EC:2.3.1.51 2.3.1.23 2.3.1.-]
CSA025168	K14457: MOGAT2, MGAT2; 2-acylglycerol O-acyltransferase 2 [EC:2.3.1.22]
CSA025169	K14457: MOGAT2, MGAT2; 2-acylglycerol O-acyltransferase 2 [EC:2.3.1.22]
CSA016717	K01074: PPT; palmitoyl-protein thioesterase [EC:3.1.2.22]
CSA028920	K01897: ACSL, fadD; long-chain acyl-CoA synthetase [EC:6.2.1.3]
CSA026812	K03921: FAB2, acyl-[acyl-carrier-protein] desaturase [EC:1.14.19.2 1.14.19.11 1.14.19.26]
CSA000990	K10781: FATB; fatty acyl-ACP thioesterase B [EC:3.1.2.14 3.1.2.21]
CSA021792	K00121: ADH5, alcohol dehydrogenase [EC:1.1.1.284 1.1.1.1]
CSA005321	K00232: ACOX1, ACOX3; acyl-CoA oxidase [EC:1.3.3.6]
CSA013107	K10781: FATB; fatty acyl-ACP thioesterase B [EC:3.1.2.14 3.1.2.21]
CSA035327	K07513: ACAA1; acetyl-CoA acyltransferase 1 [EC:2.3.1.16]
CSA034864	K01115: PLD1_2; phospholipase D1/2 [EC:3.1.4.4]
CSA001052	K13510: LPCAT1_2; lysophosphatidylcholine acyltransferase[EC:2.3.1.23 2.3.1.67]
CSA011293	K09480: DGD; digalactosyldiacylglycerol synthase [EC:2.4.1.241]
CSA015544	K10525: AOC; allene oxide cyclase [EC:5.3.99.6]
CSA019328	K05309: PTGES2; microsomal prostaglandin-E synthase 2 [EC:5.3.99.3]
CSA003686	K01115: PLD1_2; phospholipase D1/2 [EC:3.1.4.4]
CSA005815	K01115: PLD1_2; phospholipase D1/2 [EC:3.1.4.4]
CSA002136	K08730: PTDSS2; phosphatidylserine synthase 2 [EC:2.7.8.29]
CSA005774	K05894: OPR; 12-oxophytodienoic acid reductase [EC:1.3.1.42]
CSA017230	K00655: plsC; 1-acyl-sn-glycerol-3-phosphate acyltransferase [EC:2.3.1.51]
CSA030062	K01054: MGLL; acylglycerol lipase [EC:3.1.1.23]
CSA021790	K00121:ADH5,alcohol dehydrogenase [EC:1.1.1.284 1.1.1.1]
CSA029366	K05894: OPR; 12-oxophytodienoic acid reductase [EC:1.3.1.42]
CSA024872	K01114: plc; phospholipase C [EC:3.1.4.3]
CSA008570	K01115: PLD1_2; phospholipase D1/2 [EC:3.1.4.4]
CSA012038	K00128: ALDH; aldehyde dehydrogenase (NAD+) [EC:1.2.1.3]
CSA018033	K00679: phospholipid:diacylglycerol acyltransferase [EC:2.3.1.158]
CSA005692	K14156: CHK; choline/ethanolamine kinase [EC:2.7.1.32 2.7.1.82]
CSA012669	K00454: LOX2S; lipoxygenase [EC:1.13.11.12]
CSA006062	K10526: OPCL1; OPC-8:0 CoA ligase 1 [EC:6.2.1.-]
CSA006715	K18696: GDE1; glycerophosphodiester phosphodiesterase [EC:3.1.4.46]
CSA024081	K00981: CDS1, CDS2, ; phosphatidate cytidylyltransferase [EC:2.7.7.41]

Index	Compounds
pmb0165	LysoPC 16:1
pmb0852	LysoPC 18:2
pmb0854	LysoPC 18:3
pmb0855	LysoPC 16:0
pmb0864	LysoPE 14:0
pmb0866	LysoPC 14:0
pmb0874	LysoPE 18:2 (2n isomer)
pmb0876	LysoPE 16:0
pmb0882	LysoPC 18:1
pmb0883	LysoPE 18:0
pmb1562	MAG (18:4) isomer3
pmb2260	LysoPC 15:1
pmb2319	LysoPC 15:0
pmb2406	LysoPC 17:0
pmb2804	13-HPODE
pmb3121	LysoPE 18:1
pmd0132	LysoPC 16:0 (2n isomer)
pmd0151	LysoPS 22:6
pma3606	"9-Hydroxy-(10E,12Z,15Z)-octadecatrienoic acid"
pmb0149	MAG (18:4) isomer1
pmb0296	MAG (18:1) isomer2
pmb0848	LysoPC 16:1 (2n isomer)
pmb0859	LysoPC 18:1 (2n isomer)
pmb0863	LysoPC 16:2 (2n isomer)
pmb0865	LysoPC 18:3 (2n isomer)
pmb0873	LysoPC 18:2 (2n isomer)
pmb0885	"4-oxo-9Z,11Z,13E,15E-octadecatetraenoic acid"
pmb1656	MAG (18:3) isomer4
pmb2363	MAG (18:1) isomer1
pmb2388	LysoPC 18:0 (2n isomer)
pmb3132	LysoPE 18:1 (2n isomer)
pmc0960	LysoPC 20:4
pmb0161	DGMG (18:2) isomer1
pmb0163	DGMG (18:2) isomer3

geneName	metaName	R^2
CSA035088	pmb0165	0.899
CSA000654	pmb0852	-0.803
CSA000990	pmb0852	0.94
CSA001056	pmb0852	-0.843
CSA001069	pmb0852	0.86
CSA004606	pmb0852	-0.831
CSA005242	pmb0852	-0.894
CSA005266	pmb0852	0.846
CSA005325	pmb0852	0.818
CSA012072	pmb0852	0.89
CSA012667	pmb0852	-0.824
CSA013647	pmb0852	-0.841
CSA014346	pmb0852	0.914
CSA016222	pmb0852	-0.836
CSA016717	pmb0852	-0.801
CSA018764	pmb0852	-0.834
CSA019962	pmb0852	-0.806
CSA021792	pmb0852	-0.831
CSA025168	pmb0852	-0.907
CSA025169	pmb0852	-0.88
CSA026812	pmb0852	0.9
CSA028920	pmb0852	-0.842
CSA030599	pmb0852	0.939
CSA031091	pmb0852	0.921
CSA032782	pmb0852	0.89
CSA033891	pmb0852	0.811
CSA034138	pmb0852	-0.808
CSA000990	pmb0854	0.945
CSA001056	pmb0854	-0.835
CSA001069	pmb0854	0.867
CSA004606	pmb0854	-0.829
CSA005242	pmb0854	-0.865
CSA005266	pmb0854	0.836
CSA005321	pmb0854	0.802

CSA005325	pmb0854	0.818
CSA012072	pmb0854	0.856
CSA012667	pmb0854	-0.803
CSA013107	pmb0854	0.833
CSA013647	pmb0854	-0.817
CSA014346	pmb0854	0.913
CSA018764	pmb0854	-0.831
CSA021792	pmb0854	-0.802
CSA025168	pmb0854	-0.879
CSA025169	pmb0854	-0.857
CSA026812	pmb0854	0.881
CSA028920	pmb0854	-0.819
CSA030599	pmb0854	0.967
CSA031091	pmb0854	0.934
CSA032782	pmb0854	0.879
CSA000990	pmb0855	0.947
CSA001056	pmb0855	-0.831
CSA001069	pmb0855	0.873
CSA004606	pmb0855	-0.82
CSA005242	pmb0855	-0.851
CSA005266	pmb0855	0.824
CSA005325	pmb0855	0.809
CSA012072	pmb0855	0.813
CSA013107	pmb0855	0.854
CSA014346	pmb0855	0.914
CSA018764	pmb0855	-0.821
CSA025168	pmb0855	-0.869
CSA025169	pmb0855	-0.849
CSA026812	pmb0855	0.853
CSA030599	pmb0855	0.966
CSA031091	pmb0855	0.913
CSA032782	pmb0855	0.859
CSA000990	pmb0864	0.938
CSA001056	pmb0864	-0.814
CSA001069	pmb0864	0.858
CSA004606	pmb0864	-0.806
CSA005242	pmb0864	-0.852
CSA005266	pmb0864	0.816
CSA012072	pmb0864	0.839
CSA013107	pmb0864	0.806
CSA014346	pmb0864	0.903
CSA018764	pmb0864	-0.81
CSA025168	pmb0864	-0.876
CSA025169	pmb0864	-0.851
CSA026812	pmb0864	0.876
CSA030599	pmb0864	0.947
CSA031091	pmb0864	0.909
CSA032782	pmb0864	0.862
CSA000990	pmb0866	0.941
CSA001056	pmb0866	-0.824
CSA001069	pmb0866	0.879
CSA004606	pmb0866	-0.812
CSA005242	pmb0866	-0.868
CSA005266	pmb0866	0.816
CSA005321	pmb0866	0.815
CSA005325	pmb0866	0.814
CSA012072	pmb0866	0.849
CSA012667	pmb0866	-0.803
CSA013107	pmb0866	0.824
CSA013647	pmb0866	-0.807
CSA014346	pmb0866	0.911
CSA016222	pmb0866	-0.805
CSA018764	pmb0866	-0.81
CSA021792	pmb0866	-0.807
CSA025168	pmb0866	-0.893
CSA025169	pmb0866	-0.86
CSA026812	pmb0866	0.866
CSA028920	pmb0866	-0.812
CSA030599	pmb0866	0.955
CSA031091	pmb0866	0.909
CSA032782	pmb0866	0.865

CSA000654	pmb0874	-0.81
CSA000990	pmb0874	0.944
CSA001052	pmb0874	-0.809
CSA001056	pmb0874	-0.852
CSA001069	pmb0874	0.859
CSA004606	pmb0874	-0.845
CSA005242	pmb0874	-0.893
CSA005266	pmb0874	0.87
CSA005321	pmb0874	0.828
CSA005325	pmb0874	0.847
CSA011293	pmb0874	0.825
CSA012072	pmb0874	0.911
CSA012667	pmb0874	-0.836
CSA013107	pmb0874	0.822
CSA013647	pmb0874	-0.856
CSA014346	pmb0874	0.922
CSA016222	pmb0874	-0.838
CSA016717	pmb0874	-0.806
CSA018764	pmb0874	-0.847
CSA019962	pmb0874	-0.815
CSA021792	pmb0874	-0.846
CSA025168	pmb0874	-0.894
CSA025169	pmb0874	-0.875
CSA026812	pmb0874	0.913
CSA028920	pmb0874	-0.857
CSA030599	pmb0874	0.96
CSA031091	pmb0874	0.954
CSA032782	pmb0874	0.916
CSA033891	pmb0874	0.855
CSA034138	pmb0874	-0.817
CSA034864	pmb0874	0.821
CSA000990	pmb0876	0.949
CSA001056	pmb0876	-0.828
CSA001069	pmb0876	0.85
CSA004606	pmb0876	-0.818
CSA005242	pmb0876	-0.851
CSA005266	pmb0876	0.842
CSA005325	pmb0876	0.806
CSA012072	pmb0876	0.821
CSA013107	pmb0876	0.86
CSA013647	pmb0876	-0.803
CSA014346	pmb0876	0.904
CSA018764	pmb0876	-0.825
CSA025168	pmb0876	-0.871
CSA025169	pmb0876	-0.853
CSA026812	pmb0876	0.865
CSA028920	pmb0876	-0.802
CSA030599	pmb0876	0.961
CSA031091	pmb0876	0.922
CSA032782	pmb0876	0.868
CSA000654	pmb0882	-0.869
CSA000990	pmb0882	0.954
CSA001052	pmb0882	-0.808
CSA001056	pmb0882	-0.874
CSA001069	pmb0882	0.941
CSA002136	pmb0882	-0.848
CSA004606	pmb0882	-0.844
CSA005242	pmb0882	-0.944
CSA005266	pmb0882	0.859
CSA005321	pmb0882	0.869
CSA005325	pmb0882	0.896
CSA005774	pmb0882	-0.826
CSA011293	pmb0882	0.829
CSA012072	pmb0882	0.876
CSA012667	pmb0882	-0.875
CSA013647	pmb0882	-0.867
CSA014346	pmb0882	0.917
CSA016222	pmb0882	-0.888
CSA016717	pmb0882	-0.836
CSA017230	pmb0882	-0.861
CSA018764	pmb0882	-0.841

CSA019328	pmb0882	0.833
CSA019962	pmb0882	-0.828
CSA021790	pmb0882	-0.84
CSA021792	pmb0882	-0.883
CSA025168	pmb0882	-0.923
CSA025169	pmb0882	-0.896
CSA026812	pmb0882	0.932
CSA028920	pmb0882	-0.882
CSA030062	pmb0882	0.814
CSA030599	pmb0882	0.915
CSA031091	pmb0882	0.907
CSA032782	pmb0882	0.926
CSA033891	pmb0882	0.821
CSA034138	pmb0882	-0.876
CSA034864	pmb0882	0.84
CSA000990	pmb0883	0.961
CSA001052	pmb0883	-0.807
CSA001056	pmb0883	-0.856
CSA001069	pmb0883	0.859
CSA004606	pmb0883	-0.846
CSA005242	pmb0883	-0.87
CSA005266	pmb0883	0.838
CSA012072	pmb0883	0.806
CSA012667	pmb0883	-0.81
CSA013107	pmb0883	0.833
CSA013647	pmb0883	-0.821
CSA014346	pmb0883	0.897
CSA018764	pmb0883	-0.848
CSA021792	pmb0883	-0.802
CSA025168	pmb0883	-0.89
CSA025169	pmb0883	-0.867
CSA026812	pmb0883	0.864
CSA028920	pmb0883	-0.822
CSA030599	pmb0883	0.942
CSA031091	pmb0883	0.911
CSA032782	pmb0883	0.859
CSA000990	pmb1562	0.879
CSA001056	pmb1562	-0.803
CSA001069	pmb1562	0.827
CSA004606	pmb1562	-0.808
CSA014346	pmb1562	0.849
CSA018764	pmb1562	-0.808
CSA026812	pmb1562	0.845
CSA030599	pmb1562	0.886
CSA031091	pmb1562	0.88
CSA032782	pmb1562	0.806
CSA000654	pmb2260	-0.819
CSA000990	pmb2260	0.931
CSA001052	pmb2260	-0.813
CSA001056	pmb2260	-0.86
CSA001069	pmb2260	0.835
CSA004606	pmb2260	-0.838
CSA005242	pmb2260	-0.894
CSA005266	pmb2260	0.899
CSA005321	pmb2260	0.861
CSA005325	pmb2260	0.885
CSA011293	pmb2260	0.824
CSA012072	pmb2260	0.89
CSA012667	pmb2260	-0.853
CSA013107	pmb2260	0.856
CSA013647	pmb2260	-0.853
CSA014346	pmb2260	0.888
CSA016222	pmb2260	-0.838
CSA016717	pmb2260	-0.818
CSA018764	pmb2260	-0.841
CSA021790	pmb2260	-0.81
CSA021792	pmb2260	-0.872
CSA025168	pmb2260	-0.906
CSA025169	pmb2260	-0.891
CSA026812	pmb2260	0.896
CSA028920	pmb2260	-0.863

CSA030599	pmb2260	0.943
CSA031091	pmb2260	0.955
CSA032782	pmb2260	0.912
CSA033891	pmb2260	0.867
CSA034138	pmb2260	-0.842
CSA034864	pmb2260	0.843
CSA000990	pmb2319	0.94
CSA001056	pmb2319	-0.817
CSA001069	pmb2319	0.854
CSA004606	pmb2319	-0.803
CSA005242	pmb2319	-0.844
CSA005266	pmb2319	0.82
CSA012072	pmb2319	0.813
CSA013107	pmb2319	0.854
CSA014346	pmb2319	0.914
CSA018764	pmb2319	-0.806
CSA025168	pmb2319	-0.871
CSA025169	pmb2319	-0.851
CSA026812	pmb2319	0.843
CSA029366	pmb2319	0.803
CSA030599	pmb2319	0.958
CSA031091	pmb2319	0.897
CSA032782	pmb2319	0.85
CSA035088	pmb2406	0.897
CSA000990	pmb2804	0.855
CSA001069	pmb2804	0.862
CSA014346	pmb2804	0.895
CSA030599	pmb2804	0.839
CSA000654	pmb3121	-0.817
CSA000990	pmb3121	0.964
CSA001056	pmb3121	-0.845
CSA001069	pmb3121	0.87
CSA004606	pmb3121	-0.832
CSA005242	pmb3121	-0.899
CSA005266	pmb3121	0.886
CSA005321	pmb3121	0.856
CSA005325	pmb3121	0.869
CSA012072	pmb3121	0.865
CSA012667	pmb3121	-0.808
CSA013107	pmb3121	0.867
CSA013647	pmb3121	-0.843
CSA014346	pmb3121	0.917
CSA016222	pmb3121	-0.844
CSA018764	pmb3121	-0.84
CSA019962	pmb3121	-0.817
CSA021792	pmb3121	-0.829
CSA025168	pmb3121	-0.893
CSA025169	pmb3121	-0.871
CSA026812	pmb3121	0.897
CSA028920	pmb3121	-0.839
CSA030599	pmb3121	0.959
CSA031091	pmb3121	0.934
CSA032782	pmb3121	0.912
CSA033891	pmb3121	0.801
CSA034138	pmb3121	-0.807
CSA000990	pmd0132	0.897
CSA001069	pmd0132	0.869
CSA012072	pmd0132	0.814
CSA013107	pmd0132	0.842
CSA014346	pmd0132	0.907
CSA025168	pmd0132	-0.809
CSA026812	pmd0132	0.813
CSA030599	pmd0132	0.962
CSA031091	pmd0132	0.889
CSA032782	pmd0132	0.832
CSA000990	pmd0151	0.856
CSA001069	pmd0151	0.826
CSA014346	pmd0151	0.821
CSA030599	pmd0151	0.9
CSA000990	pma3606	0.92
CSA001056	pma3606	-0.847

CSA001069	pma3606	0.814
CSA005242	pma3606	-0.829
CSA005266	pma3606	0.845
CSA013107	pma3606	0.853
CSA014346	pma3606	0.9
CSA018764	pma3606	-0.801
CSA025168	pma3606	-0.883
CSA025169	pma3606	-0.884
CSA029366	pma3606	0.841
CSA030599	pma3606	0.906
CSA031091	pma3606	0.847
CSA032782	pma3606	0.81
CSA000990	pmb0149	0.885
CSA001052	pmb0149	-0.803
CSA001056	pmb0149	-0.836
CSA001069	pmb0149	0.845
CSA004606	pmb0149	-0.834
CSA005242	pmb0149	-0.864
CSA005266	pmb0149	0.85
CSA005325	pmb0149	0.904
CSA011293	pmb0149	0.808
CSA013647	pmb0149	-0.814
CSA014346	pmb0149	0.826
CSA018764	pmb0149	-0.84
CSA026812	pmb0149	0.839
CSA028920	pmb0149	-0.819
CSA030062	pmb0149	0.869
CSA030599	pmb0149	0.895
CSA031091	pmb0149	0.905
CSA032782	pmb0149	0.872
CSA000654	pmb0296	-0.917
CSA000990	pmb0296	0.842
CSA001052	pmb0296	-0.806
CSA001056	pmb0296	-0.827
CSA002136	pmb0296	-0.877
CSA004606	pmb0296	-0.815
CSA005242	pmb0296	-0.937
CSA005266	pmb0296	0.839
CSA005325	pmb0296	0.818
CSA005774	pmb0296	-0.88
CSA011293	pmb0296	0.812
CSA012038	pmb0296	-0.881
CSA012072	pmb0296	0.897
CSA012667	pmb0296	-0.851
CSA013647	pmb0296	-0.903
CSA016222	pmb0296	-0.943
CSA016717	pmb0296	-0.849
CSA017230	pmb0296	-0.864
CSA018033	pmb0296	0.808
CSA018764	pmb0296	-0.809
CSA019328	pmb0296	0.904
CSA019962	pmb0296	-0.897
CSA021790	pmb0296	-0.875
CSA021792	pmb0296	-0.912
CSA024872	pmb0296	-0.817
CSA025168	pmb0296	-0.894
CSA025169	pmb0296	-0.854
CSA026812	pmb0296	0.834
CSA028920	pmb0296	-0.897
CSA032782	pmb0296	0.855
CSA033891	pmb0296	0.843
CSA034138	pmb0296	-0.847
CSA034864	pmb0296	0.884
CSA000654	pmb0848	-0.872
CSA000990	pmb0848	0.976
CSA001052	pmb0848	-0.876
CSA001056	pmb0848	-0.916
CSA001069	pmb0848	0.835
CSA002136	pmb0848	-0.831
CSA004606	pmb0848	-0.903
CSA005242	pmb0848	-0.946

CSA005266	pmb0848	0.9
CSA005321	pmb0848	0.819
CSA005325	pmb0848	0.818
CSA005692	pmb0848	-0.81
CSA005774	pmb0848	-0.803
CSA006062	pmb0848	-0.832
CSA011293	pmb0848	0.829
CSA012038	pmb0848	-0.873
CSA012072	pmb0848	0.877
CSA012667	pmb0848	-0.892
CSA012669	pmb0848	-0.817
CSA013107	pmb0848	0.805
CSA013647	pmb0848	-0.927
CSA014346	pmb0848	0.904
CSA016222	pmb0848	-0.893
CSA016717	pmb0848	-0.887
CSA017230	pmb0848	-0.813
CSA018764	pmb0848	-0.899
CSA019962	pmb0848	-0.863
CSA021790	pmb0848	-0.86
CSA021792	pmb0848	-0.909
CSA025168	pmb0848	-0.927
CSA025169	pmb0848	-0.903
CSA026812	pmb0848	0.9
CSA028920	pmb0848	-0.918
CSA030599	pmb0848	0.883
CSA031091	pmb0848	0.903
CSA032782	pmb0848	0.911
CSA033891	pmb0848	0.82
CSA034138	pmb0848	-0.851
CSA034864	pmb0848	0.854
CSA000654	pmb0859	-0.865
CSA000990	pmb0859	0.941
CSA001056	pmb0859	-0.836
CSA001069	pmb0859	0.901
CSA002136	pmb0859	-0.845
CSA004606	pmb0859	-0.814
CSA005242	pmb0859	-0.931
CSA005266	pmb0859	0.839
CSA005321	pmb0859	0.834
CSA005325	pmb0859	0.856
CSA005774	pmb0859	-0.828
CSA012038	pmb0859	-0.804
CSA012072	pmb0859	0.819
CSA012667	pmb0859	-0.82
CSA013647	pmb0859	-0.842
CSA014346	pmb0859	0.864
CSA016222	pmb0859	-0.888
CSA017230	pmb0859	-0.84
CSA018764	pmb0859	-0.818
CSA019328	pmb0859	0.853
CSA019962	pmb0859	-0.848
CSA021792	pmb0859	-0.84
CSA025168	pmb0859	-0.896
CSA025169	pmb0859	-0.861
CSA026812	pmb0859	0.905
CSA028920	pmb0859	-0.849
CSA030062	pmb0859	0.809
CSA030599	pmb0859	0.853
CSA031091	pmb0859	0.849
CSA032782	pmb0859	0.891
CSA034138	pmb0859	-0.825
CSA000990	pmb0863	0.926
CSA001056	pmb0863	-0.831
CSA001069	pmb0863	0.835
CSA005242	pmb0863	-0.871
CSA012667	pmb0863	-0.813
CSA013647	pmb0863	-0.806
CSA014346	pmb0863	0.88
CSA016222	pmb0863	-0.813
CSA021792	pmb0863	-0.807

CSA025168	pmb0863	-0.92
CSA025169	pmb0863	-0.89
CSA026812	pmb0863	0.839
CSA028920	pmb0863	-0.809
CSA030599	pmb0863	0.863
CSA031091	pmb0863	0.82
CSA032782	pmb0863	0.811
CSA019962	pmb0865	-0.831
CSA000654	pmb0873	-0.889
CSA000990	pmb0873	0.921
CSA001052	pmb0873	-0.811
CSA001056	pmb0873	-0.844
CSA001069	pmb0873	0.856
CSA002136	pmb0873	-0.851
CSA004606	pmb0873	-0.841
CSA005242	pmb0873	-0.943
CSA005266	pmb0873	0.823
CSA005321	pmb0873	0.821
CSA005325	pmb0873	0.835
CSA005774	pmb0873	-0.842
CSA011293	pmb0873	0.81
CSA012038	pmb0873	-0.864
CSA012072	pmb0873	0.885
CSA012667	pmb0873	-0.858
CSA013647	pmb0873	-0.874
CSA014346	pmb0873	0.83
CSA016222	pmb0873	-0.913
CSA016717	pmb0873	-0.83
CSA017230	pmb0873	-0.846
CSA018764	pmb0873	-0.833
CSA019328	pmb0873	0.867
CSA019962	pmb0873	-0.894
CSA021790	pmb0873	-0.83
CSA021792	pmb0873	-0.888
CSA025168	pmb0873	-0.924
CSA025169	pmb0873	-0.87
CSA026812	pmb0873	0.901
CSA028920	pmb0873	-0.887
CSA030599	pmb0873	0.838
CSA031091	pmb0873	0.866
CSA032782	pmb0873	0.88
CSA034138	pmb0873	-0.833
CSA034864	pmb0873	0.857
CSA000990	pmb0885	0.909
CSA001052	pmb0885	-0.803
CSA001056	pmb0885	-0.854
CSA004606	pmb0885	-0.829
CSA005266	pmb0885	0.825
CSA013107	pmb0885	0.811
CSA014346	pmb0885	0.822
CSA018764	pmb0885	-0.846
CSA025168	pmb0885	-0.815
CSA025169	pmb0885	-0.844
CSA030599	pmb0885	0.885
CSA031091	pmb0885	0.865
CSA000990	pmb1656	-0.826
CSA001069	pmb1656	-0.817
CSA005242	pmb1656	0.802
CSA014346	pmb1656	-0.851
CSA025168	pmb1656	0.827
CSA025169	pmb1656	0.866
CSA034138	pmb1656	0.815
CSA000654	pmb2363	-0.942
CSA000990	pmb2363	0.882
CSA001052	pmb2363	-0.818
CSA001056	pmb2363	-0.855
CSA002136	pmb2363	-0.906
CSA004606	pmb2363	-0.831
CSA005242	pmb2363	-0.961
CSA005266	pmb2363	0.879
CSA005321	pmb2363	0.843

CSA005325	pmb2363	0.86
CSA005774	pmb2363	-0.906
CSA011293	pmb2363	0.817
CSA012038	pmb2363	-0.88
CSA012072	pmb2363	0.898
CSA012667	pmb2363	-0.871
CSA013647	pmb2363	-0.92
CSA014346	pmb2363	0.835
CSA016222	pmb2363	-0.964
CSA016717	pmb2363	-0.867
CSA017230	pmb2363	-0.892
CSA018033	pmb2363	0.8
CSA018764	pmb2363	-0.827
CSA019328	pmb2363	0.902
CSA019962	pmb2363	-0.9
CSA021790	pmb2363	-0.899
CSA021792	pmb2363	-0.933
CSA024081	pmb2363	-0.811
CSA024872	pmb2363	-0.842
CSA025168	pmb2363	-0.92
CSA025169	pmb2363	-0.885
CSA026812	pmb2363	0.856
CSA028920	pmb2363	-0.914
CSA031091	pmb2363	0.815
CSA032782	pmb2363	0.887
CSA033891	pmb2363	0.851
CSA034138	pmb2363	-0.881
CSA034864	pmb2363	0.886
CSA000654	pmb2388	-0.809
CSA000990	pmb2388	0.867
CSA001069	pmb2388	0.822
CSA002136	pmb2388	-0.814
CSA005242	pmb2388	-0.863
CSA014346	pmb2388	0.822
CSA016222	pmb2388	-0.841
CSA019328	pmb2388	0.87
CSA025168	pmb2388	-0.863
CSA025169	pmb2388	-0.814
CSA026812	pmb2388	0.848
CSA032782	pmb2388	0.811
CSA000654	pmb3132	-0.902
CSA000990	pmb3132	0.823
CSA002136	pmb3132	-0.874
CSA005242	pmb3132	-0.861
CSA005321	pmb3132	0.847
CSA005774	pmb3132	-0.874
CSA012072	pmb3132	0.913
CSA013647	pmb3132	-0.817
CSA016222	pmb3132	-0.906
CSA017230	pmb3132	-0.855
CSA019328	pmb3132	0.84
CSA019962	pmb3132	-0.897
CSA021790	pmb3132	-0.8
CSA021792	pmb3132	-0.836
CSA026812	pmb3132	0.837
CSA028920	pmb3132	-0.802
CSA032782	pmb3132	0.855
CSA033891	pmb3132	0.809
CSA034864	pmb3132	0.853
CSA000654	pmc0960	-0.872
CSA000990	pmc0960	0.859
CSA001069	pmc0960	0.859
CSA002136	pmc0960	-0.845
CSA005242	pmc0960	-0.913
CSA005325	pmc0960	0.847
CSA005774	pmc0960	-0.844
CSA012038	pmc0960	-0.824
CSA012072	pmc0960	0.834
CSA013647	pmc0960	-0.826
CSA016222	pmc0960	-0.896
CSA017230	pmc0960	-0.845

CSA019328	pmc0960	0.901
CSA019962	pmc0960	-0.889
CSA021792	pmc0960	-0.824
CSA025168	pmc0960	-0.836
CSA026812	pmc0960	0.886
CSA028920	pmc0960	-0.838
CSA030062	pmc0960	0.826
CSA031091	pmc0960	0.802
CSA032782	pmc0960	0.865
CSA034864	pmc0960	0.81
CSA005242	pmb0161	-0.809
CSA012072	pmb0161	0.84
CSA016222	pmb0161	-0.817
CSA021792	pmb0161	-0.837
CSA025168	pmb0161	-0.836
CSA033891	pmb0161	0.806
CSA034864	pmb0161	0.838
CSA000654	pmb0163	-0.813
CSA005242	pmb0163	-0.832
CSA012038	pmb0163	-0.821
CSA012072	pmb0163	0.836
CSA013647	pmb0163	-0.819
CSA016222	pmb0163	-0.838
CSA021790	pmb0163	-0.817
CSA021792	pmb0163	-0.862
CSA025168	pmb0163	-0.847
CSA028920	pmb0163	-0.824
CSA033891	pmb0163	0.812
CSA034864	pmb0163	0.857
