# Supplementary material for: Exogenous abscisic acid induces the lipid and flavonoid metabolism of tea plants under drought stress
Source: Sci Rep. 2020 Jul 23;10:12275. doi: 10.1038/s41598-020-69080-1 (PMC7378251; doi:10.1038/s41598-020-69080-1)
Supplement: Supplementary file 9 — Supplementary table S6. [file 41598_2020_69080_MOESM9_ESM.pdf]

Exogenous abscisic acid induces the lipid and flavonoid metabolism of tea plants under drought stress

Zhongshuai Gai [12#], Yu Wang[1#], Yiqian Ding[1], Wenjun Qian[1], Chen Qiu[1], Hui Xie[1], Litao Sun[1], Zhongwu Jiang[2], Qingping Ma[3], Linjun Wang[4], Zhaotang Ding[1]*

[1]Tea Research Institute, Qingdao Agricultural University, Qingdao 266109, China

[2]College of Life Science, Yantai University, Yantai, Shandong, 264005, China

[3]College of agriculture, Liaocheng University, Liaocheng, Shandong, 252059, China

[4]Fruit tea station of weihai agricultural and rural affairs service center, Weihai, Shandong, 264200, China

# These authors contributed equally to this study.

***CORRESPONDENCE:** Dr Zhaotang Ding E-mails: dzttea@163.com

Table S6 Primer used for qRT-PCR

Gene ID	Forward primer	Reverse primer
CSA030599	TGCGGGCTATGATTGTGACTAC	ACATCTTCGCCTGGATTAGGG
CSA011597	TCTGCTGATAGCTCCACAGGG	GGACAAGGCTTTTGAAAACACTTC
CSA032782	TTCTTCTCCACCAAAACCACG	CAGCAATCATCAATGCCGACT
CSA000990	GCTGCAATCCCTAACCAAAGTATC	AGCTGACCAAGGCTCCCAAG
CSA016635	TTTACAGGGTCCACGGAAGTAGG	ACCGCCCAAAAGAGCAAGAT
CSA026735	CTTGGAGCAAGTTGTCGAGTTTT	AGGCTGTTGGCTAAGCAGGA
CSA003949	AAACCTATCCCTGCCTCGTCG	CACAAGTCTCCCACATCGTACTCA
CSA000931	ATCACCCCGAGCCCTAACTG	TTGAAGAAAGCGAAGAAGCACTAA
CSA033689	CCTTGGGTCTGGTGGGACTAT	CAACTTGAGCATCTTGCTCTTCTT
CSA020560	GATTGGTCGGGATGGGAGTG	CCGACAAATCTATGCGAGGCT
GADPH	TTGGCATCGTTGAGGGTCT	CAGTGGGAACACGGAAAGC
